# Modeling of the Electrostatic Interaction and Catalytic Activity of [NiFe] Hydrogenases on a Planar Electrode

**DOI:** 10.1021/acs.jpcb.2c05371

**Published:** 2022-10-21

**Authors:** Manuel Antonio Ruiz-Rodríguez, Christopher D. Cooper, Walter Rocchia, Mosè Casalegno, Yossef López de los Santos, Guido Raos

**Affiliations:** † Dipartimento di Chimica, Materiali e Ingegneria Chimica “G. Natta”, Politecnico di Milano, 20133Milano, Italy; ‡ Department of Mechanical Engineering and Centro Científico Tecnológico de Valparaíso, Universidad Técnica Federico Santa María, Valparaíso, 2340000, Chile; § CONCEPT Lab, Istituto Italiano di Tecnologia, 16163Genova, Italy; ∥ Centre Armand-Frappier Santé, Biotechnologie, Institut national de la recherche scientifique (INRS), Université du Québec, Laval, QuébecHV7 1B7, Canada

## Abstract

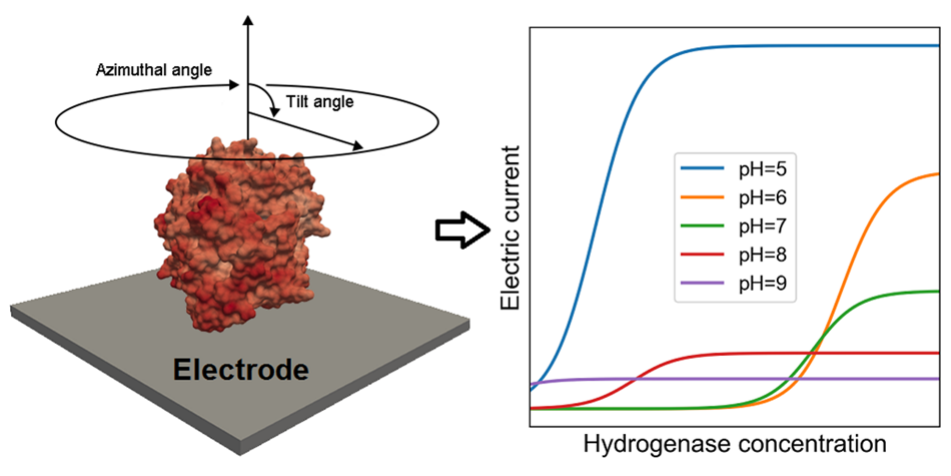

Hydrogenases are a group of enzymes that have caught the interest of researchers in renewable energies, due to their ability to catalyze the redox reaction of hydrogen. The exploitation of hydrogenases in electrochemical devices requires their immobilization on the surface of suitable electrodes, such as graphite. The orientation of the enzyme on the electrode is important to ensure a good flux of electrons to the catalytic center, through an array of iron–sulfur clusters. Here we present a computational approach to determine the possible orientations of a [NiFe] hydrogenase (PDB 1e3d) on a planar electrode, as a function of pH, salinity, and electrode potential. The calculations are based on the solution of the linearized Poisson–Boltzmann equation, using the PyGBe software. The results reveal that electrostatic interactions do not truly immobilize the enzyme on the surface of the electrode, but there is instead a dynamic equilibrium between different orientations. Nonetheless, after averaging over all thermally accessible orientations, we find significant differences related to the solution’s salinity and pH, while the effect of the electrode potential is relatively weak. We also combine models for the protein adsoption–desorption equilibria and for the electron transfer between the proteins and the electrode to arrive at a prediction of the electrode’s activity as a function of the enzyme concentration.

## Introduction

Hydrogenases are a family of enzymes that catalyze the redox reaction of hydrogen. During the oxidation of hydrogen, two electrons are removed from the hydrogen molecule, generating two protons. In the reduction pathway, two electrons are added to two protons, generating molecular hydrogen:^1−3^
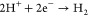


Hydrogenases are found in bacteria, archaea, and some eukarya, living in a wide spectrum of environments: aerobic, anaerobic,^1^ or extreme conditions like hydrothermal vents.^4^ Their role is to oxidize H_2_ to H^+^ or reduce H^+^ to H_2_, thus providing a reversible route for energy conversion depending on the cell’s necessities. Hydrogenases coupled to other enzymes play a major role in the fermentation of biological substances to CH_4_ and in microbial phosphorylation, where H_2_ can serve as an energy source in place of NADH. In other metabolic routes, hydrogenases generate molecular hydrogen as a subproduct of reductive processes.^1^ Depending on the organism, hydrogenases can be found floating free in the cytoplasm, associated with the cellular membrane, or in the periplasm of the cell as part of a reaction chain involving other proteins. In eukaryotic cells, they are often located in specialized compartments.^1,5^

**Figure 1:**
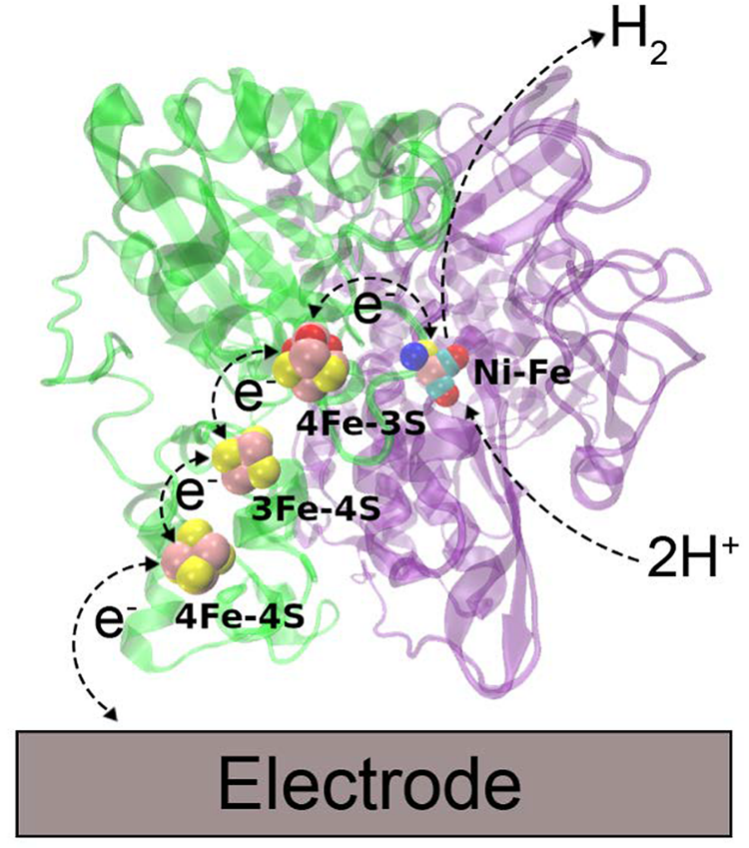
Model of the *Desulfovibrio desulfurincans* hydrogenase (1e3d) on an electrode. The protein’s large and small subunits are depicted in purple and green, respectively. The three iron–sulfur clusters and the catalytic center are represented as follows: sulfur (yellow), iron (pink), oxygen (red), nickel (blue), and carbon (turquoise). The external iron–sulfur cluster is the entry point for electron transfer with the electrode.

Hydrogenases can be categorized into three major groups, depending on the composition of the catalytic center. The catalytic center of [NiFe] hydrogenases (Figure 1) consists of a heteronuclear core containing these two metals coordinated with cysteines, one −CN group, and two −CO groups.^6^ A subclass of this group collects the so-called [NiFeSe] hydrogenases, which contains the same heteronuclear core but with the substitution of cysteine by selenocysteine within the catalytic center. The second class comprises [FeFe] hydrogenases, containing two iron atoms within the catalytic center, which is connected to a [4Fe4S] cluster. A third class comprises [Fe]-only hydrogenases. These enzymes have the peculiarity of having a single Fe atom within their catalytic center.^1,7^

In recent years, hydrogenases have captured the interest of researchers due to their possible applications in the field of renewable energies.^3^ Great efforts have been made in order to understand their structures and catalytic mechanisms, and how they could be applied to energy conversion. One of these approaches has been the incorporation of hydrogenases on the anode in fuel cells to produce electricity, or on the cathode of (photo)electrochemical cells to produce hydrogen, avoiding the use of rare, expensive, and poison-prone metals such as platinum.^8−10^ Some researchers have addressed the use of immobilized hydrogenases directly on electrodes made of a diversity of materials, testing their performance in titanium,^9,11,12^ gold,^4,13,14^ silver,^15^ or graphite^16^ with different degrees of success. Other studies have explored the possibility of increasing the effective surface of the electrodes using porous carbon materials^16−18^ or nanoparticles.^13^

The diverse degrees of success in the experimental studies indicate that several factors, other than the choice of the enzyme and the substrate, could be involved in the rate of reaction.^3,5^ Several authors have pointed out^19−21^ that the orientation of the enzymes on the surface of the electrode has a direct effect on the reaction rate. Indeed, a whole review has been published recently on this topic.^22^ As illustrated in Figure 1, the best orientations are believed to be those in which the external iron–sulfur cluster is as close as possible to the electrode. This favors the transfer of electrons from the electrode to the catalytic center, through the array of iron–sulfur clusters that works as a conducting wire. Note that the catalytic center is often protected from the exterior of the protein, as it resides within a pocket that prevents direct electron transfer to it.^1^ For less favorable orientations, the electron transfer rate would determine the overall rate for the production or consumption of hydrogen, reducing the effectiveness of the enzyme.

Depending on whether the functionalization of the electrode is achieved by physical adsorption or covalent attachment, the protein orientation can be considered to be either reversible, and therefore satisfying an equilibrium condition, or irreversibly fixed at the moment of attachment. In turn, physical adsorption generally involves hydrogen bonding, van der Waals, hydrophobic, and electrostatic interactions. Electrostatic effects can be a dominant effect for the orientation of immobilized proteins,^23−26^ even though the other factors may have a strong effect on the overall (orientationally averaged) protein–electrode interaction energy.^27^ Electrostatic interactions between a protein and a conducting surface have sometimes been described in terms of the dipole moment of the isolated protein (i.e., without the electrode) that may be readily extracted from molecular dynamics simulations.^23^ Within this scheme, the protein should preferentially absorb with its dipole orthogonal to the surface. However, the dipole provides only a very rough description of a complex charge distribution, which may fail at close range. The orientation may thus depend also on local effects, connected to the presence of specific functional groups or “patches” on the surface of the electrode or of the enzyme.^28^ We shall return to this point below, in the discussion of our results.

Electrostatic effects in biomolecules and electrolyte solutions can be modeled by a range of methods, each with its strengths and weaknesses in terms of simplicity, generality, and computational cost.^29^ One approach that balances these contrasting requirements is the one based on the Poisson–Boltzmann equation, which describes the distribution of mobile ions and the electrostatic potential around one or more charged objects, such as a protein, an electrode, or their combination.^30,31^ Several software tools solve this equation numerically, including for example APBS,^32^ Delphi,^33,34^ MEAD,^35^ MIBPB,^36^ AFMPB,^37^ and TABI.^38^ Here we have selected PyGBe,^39−41^ due to its ability to calculate efficiently the electrostatic interaction between multiple bodies at close range.

The present study uses computational simulations with PyGBe to calculate the electrostatic component of the interaction between a [NiFe] hydrogenase and an electrode, as a function of orientation, pH, salinity, and electric potential. The key question we attempt to answer is whether it is possible to immobilize and control the orientation of the enzyme on the surface of an electrode by tuning these variables, without modifying the chemistry of the electrode (e.g., by a self-assembled monolayer of functional molecules or by covalent bonding of the protein to it) or of the protein (e.g., by introducing selected point mutations of the amino acids at its surface, away from the active site). We also offer a prediction of the activity of the functionalized electrodes as a function of the enzyme concentration, based on simple assumptions about the adsorption equilibria and the electron transfer between the proteins and the electrodes.

## Systems and Computational Methods

### The Protein

The hydrogenase used in this study is a [NiFe] hydrogenase isolated from *Desulfovibrio desulfuricans* ATCC 27774.^42^ This hydrogenase can be found in the periplasm of the cell associated with the periplasmic tetraheme cytochrome *c*_3_, taking care of the first step toward recycling the chemical energy liberated during the redox reaction of hydrogen back to the cytoplasm. It is a heterodimer of 89 kDa, with two subunits with molecular masses of 62 and 27 kDa. It contains an array of three iron–sulfur clusters: two [4Fe4S] clusters and one [3Fe4S] cluster, respectively, denominated distal (or external), proximal, and medial clusters. The Ni and Fe atoms within the catalytic core are coordinated by one −CN group, two −CO groups, and four cysteines.^42^ This specific hydrogenase was chosen for our study mainly because of the good resolution in the published crystal structure (1.80 Å).

The crystal structure of the [NiFe] hydrogenase was obtained from the Protein Data Bank^43^ as a PDB file (1e3d). This file contains the crystallized structure of the hydrogenase in the form of a tetramer, together with the solvent molecules and ions associated with the hydrogenase at the moment of crystallization. Thus, we preprocess the PDB file with the pdbeditor software,^43^ in order to isolate the system to be used for the calculations. This includes only the A chain (small subunit, 27 kDa) and the B chain (big subunit, 62 kDa), without the water molecules and ions.

### The Physical Model

**Figure 2:**
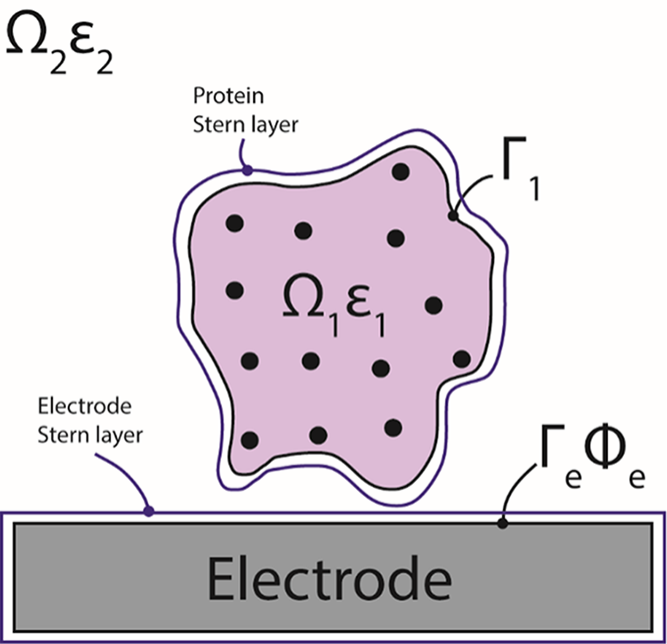
Electrostatic model of the protein–electrode interaction. Region Ω_1_ corresponds to the protein, with fixed point charges (black dots). It is bounded by a surface Γ_1_ and a Stern layer. Region Ω_2_ corresponds to the electrolyte solution. Surface Γ_e_ corresponds to the boundary of the electrode, also surrounded by a Stern layer.

The calculations of the interaction between the protein and the electrode are based on the linearized Poisson–Boltzmann equation. The enzyme is assumed to be rigid, in a conformation identical to that extracted from the crystal. It is immersed in an implicit solvent, consisting of water and mobile salt ions.^29−31^ Fixed point charges are arranged inside the protein, at the positions of the atoms. The interior and exterior of the protein are defined by the solvent-excluded surface (SES). The relative permittivity (or dielectric constant) considered for the inside of the protein is ϵ_1_ = 4, while the solvent region has the relative permittivity of water (ϵ_2_ = 80). The electrode is modeled as a conductor with the geometry of a rectangular cuboid with dimensions 250 × 250 × 10 Å^3^. In a preliminary set of calculations, we checked that the calculated interaction energies do not change significantly upon further increases in the size of the electrode. The electrode behaves as a metallic conductor and does not have an associated permittivity, but its electrostatic potential has a fixed value at all points of a fine mesh representing its surface. The model is illustrated schematically in Figure 2.

The model described in Figure 2 leads to a system of partial differential equations that were solved numerically with the PyGBe program.^39−41^ PyGBe—pronounced “pig-bee”—is based on a library of routines written in CUDA and exploiting the parallelism afforded by graphical processing units (GPUs), which can be driven by user-modifiable Python scripts. It was explicitly developed to calculate the electrostatic interaction between multiple bodies. It uses a boundary element approach to obtain the electrostatic potential ϕ(**r**), by solving the Poisson equation inside the protein (region Ω_1_) and the linearized Poisson–Boltzmann equation in the surrounding solvent (region Ω_2_):
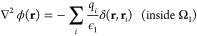
1
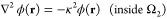
2

The summation in eq 1 runs over all the protein’s atomic charges, while κ is the inverse of the Debye–Hückel screening length, which depends on the overall concentration of small ions dissolved in the aqueous medium:
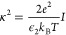
3a
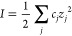
3bwhere *c*_*j*_ and *q*_*j*_ = *ez*_*j*_ are the concentrations and charges of the ions, *k*_B_*T* is the thermal energy, and *e* is the elementary charge. The ionic strength *I* coincides with the salt concentration, for a monovalent salt such as NaCl (*z*_*j*_ = ±1). For a physiological solution with *I* = 0.15 mol/L, one has κ = 0.125 Å^–1^ at room temperature. In addition, we have also considered a salt-free aqueous solution with *I* = 0.0 mol/L (neglecting the small contributions coming from H^+^ and OH^–^ ions). Equations 1 and 2 are coupled, because ϕ(**r**) and the electric displacement [−ϵ_r_∇ϕ(**r**)] must be continuous at the interface between Ω_1_ and Ω_2_ (i.e., on the protein’s SES). In addition, on the electrode’s surface (Γ_e_) we adopted a Dirichlet boundary condition, whereby the potential takes a constant value ϕ_e_:
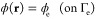
4while the electric displacement at the water–electrode interface (more precisely, its component along the normal direction **n**) gives the local charge density (unit per area). Unlike the potential, which is constant throughout the electrode, this may depend on the position **r**:
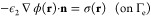
5where σ(**r**) is the induced surface electric charge density.

In the calculations we included two ion-exclusion layers (denominated “Stern layers” within this study), respectively surrounding the protein and the electrode. Each layer corresponds to a region with a thickness of 2.0 Å, which has the dielectric constant of water (ϵ_2_ = 80) but a local ion concentration equal to zero. The purpose of this layer is to prevent excessive accumulation of positive/negative ions in regions with very negative/positive potentials. It is essentially an empirical correction for the assumption inherent to the Poisson–Boltzmann model of electrolyte solutions, without any short-range correlations related to the size of the ions (assumed to be pointlike). The distance between the surface of the electrode and the van der Waals surface of the closest atom was kept at a constant value of 4.1 Å, in order to avoid overlap between their Stern layers. Below this distance, the continuum hypothesis that underlies the Poisson–Boltzmann equation might be questionable. Since we did not optimize it, include other contributions to the energy, or allow changes in the protein structure, our calculations are likely to underestimate the strength of the protein–electrode interactions.

To better understand how different factors could affect the interaction of the hydrogenase with the electrode, a computational experiment was designed considering the following factors: solution pH (5, 6, 7, 8, and 9), electric potential of the electrode ϕ_e_ (−0.05, 0.00, and 0.05 V), and salt concentration in the solution (*I* = 0.15 or 0.0 M). Note that the pH is limited to a range of values where protein denaturation is not expected to occur, while the electrostatic potential of the electrode is limited by the range of validity of the linearized Poisson–Boltzmann approach (|*eϕ*_e_| < *k*_B_*T*, where *e* is the elementary charge and *k*_B_*T* is the thermal energy). Note that ϕ_e_ is an “electric potential” and its value is zero at infinity. It is not an “electrode potential” measured against some reference electrode.^44^

### Electrostatic Adsorption Energies

To calculate the electrostatic interaction between the protein and the electrode, it is first necessary to assign the proper charge and radius to the atoms, according to a specific force field, and define the surface of the protein. For this purpose, a series of “pqr” files containing the Cartesian coordinates of the atoms, their charges, and van der Waals radii were created using the pbd2pqr software.^45^

One of the problems in assigning the atomic charges in hydrogenases is the presence of nonstandard atomic groups, with transition metal ions within the iron–sulfur clusters and in the catalytic centers of these enzymes. We determined their charges by quantum chemical calculations on the iron–sulfur clusters and the catalytic center extracted from the enzyme, where the dangling chemical bonds were saturated with CH_3_ groups. These atom selections were used to calculate the CHELPG charges,^46^ based on single-point unrestricted density function theory (DFT) calculations using the ORCA software.^47,48^ A Gaussian basis set (def2-SVP)^49^ was selected to perform the calculations with a tight self-consistent-field option, using the PBE0^50^ hybrid density functional to compute the exchange–correlation energy. We performed calculations for the catalytic center with a total charge of 0 and spin multiplicities of 1, 3, 5, 7, and 9; for the external cluster, a total charge of 0 and multiplicities of 1, 3, 5, 7, and 9; for the medial cluster, a total charge of +2 and multiplicities of 2, 4, 6, 8, and 10; and for the proximal cluster, a total charge of −3 and multiplicities of 1, 3, 5, 7, and 9. In each case, we adopted the CHELPG charges corresponding to the spin multiplicity with the lowest energy. All these data are reported in the Supporting Information.

The Amber^46^ force field file, used by pdb2pqr to create the final pqr files, was modified by adding the calculated CHELPG charges. The pdb2pqr program was then run using standard settings for the “propka” method.^45^ This assigns the protonation state of the protein’s ionizable groups, depending on the value of the solution pH. This protonation state was assumed to be fixed, independently of the distance and orientation of the protein on the electrode. In principle, this assumption could be relaxed, with an increase in calculation time.^24^ The total charge on the protein and the modulus of its dipole moment are reported in Table 1, for each pH value. Note that the dipole moment of an object with nonzero charge depends on the choice of pole for its evaluation. Our values have been calculated with respect to the center of charge of the protein.

**Table 1:** Total Charge on the Protein and Modulus of Its Dipole Moment, at Each pH Value^a^

pH	charge (*e*)	dipole (*e* Å)
5	15.6	1495.8
6	3.6	1497.5
7	–5.4	1500.6
8	–12.4	1494.3
9	–15.4	1504.0

a The elementary charge is *e* = 1.602 × 10^–19^ C. For the dipoles, 1 *e* Å = 4.803 D.

Simulations in PyGBe require that information about the protein structure is transferred to a mesh representing its surface. The mesh files for our calculations were created from the pqr files using Nanoshaper.^51^ The following settings were adopted, seeking a balance between cost and numerical accuracy of the calculations: “grid scale” = 2.0, “smooth mesh” = true, “probe radius” = 1.4, and “keep water shaped cavities” = true. The probe radius of 1.4 Å is the standard value for a water molecule. This is considered as a sphere rolling around the protein, thus generating its SES. Example input files for Nanoshaper can be found in the Supporting Information.

Having defined the atomic charges and the meshes representing the protein and electrode surfaces, the PyGBe program was used to solve the linearized Poisson–Boltzmann equation.^39−41^ The solution provided by PyGBe consists of the values of the electrostatic potential and the normal component of its gradient, for each grid point of each surface. These can then be used to obtain the electrostatic component of the free energy of the system. According to our assumption of reversible adsorption equilibrium, the free energy determines the probability that the enzyme adopts a specific orientation on the electrode.

In our study we considered the electrostatic component of the free energy of the system as the total energy of the hydrogenase–electrode system. This is calculated by PyGBe as the sum of Coulomb, solvation, and surface contributions, according to the equation
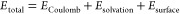
6

The Coulomb energy is calculated from the Coulomb interactions of all point charges:
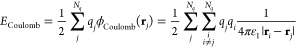
7where ε_1_ is the dielectric constant within the protein, ϕ_Coulomb_(**r**_*j*_) is the Coulomb potential at the position charge *q*_*j*_ due to the other charges (*q*_*i*_), and the double summation runs over all the protein’s charged atoms. This can be a large but constant term, being independent of the protein’s orientation and distance from the electrode.

The solvation energy is the energy contribution from the protein’s surroundings: solvent polarization, charged surfaces, and possibly other proteins. It is calculated as
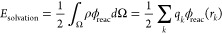
8where ρ is the charge distribution and ϕ_reac_ = ϕ – ϕ_Coulomb_ is the electrostatic potential that arises due to the solvent’s reaction, that contains the contribution of the polarization of the solute and of the solvent. Again, the summation runs over all the protein’s atomic charges.

Finally, the surface energy is
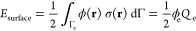
9where the integral is performed over the electrode’s surface and *Q*_e_ is the net charge on it.

The interaction energy is calculated by subtracting the values of the energy of the isolated electrode and the isolated protein from the total energy:
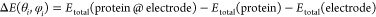
10

The values of the total energies for the protein and the electrode were calculated separately with PyGBe, using the same meshes of the combined calculation. A negative value of the interaction energy means the adsorbed state (protein@electrode) is energetically more stable than the desorbed state. The (θ_*i*_, φ_*i*_) arguments appearing on the left-hand side of eq 10 emphasize that this energy depends on the protein orientation, to be discussed under Protein Orientations and Probabilities.

### Protein Orientations and Probabilities

**Figure 3:**
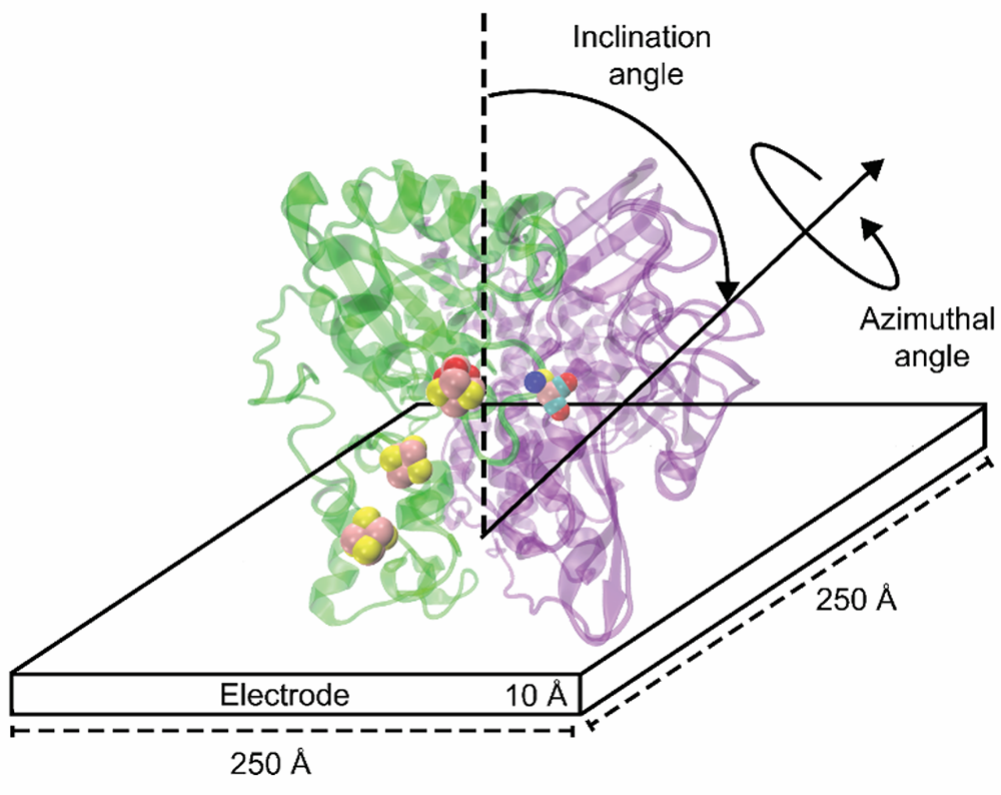
Model used to test the protein–electrode electrostatic interaction, defining the tilt (θ) and azimuthal angles (φ).

Using PyGBe, we calculated the total energy for different orientations of the hydrogenase on the electrode. As shown in Figure 3, the orientation is defined by an inclination or tilt angle θ (0° ≤ θ ≤ 180°) and an azimuthal angle φ (0° ≤ φ < 180°).^41^

In order to have an even and exhaustive sampling of the orientation, the tilt angle was incremented in even steps of dθ = 10°, while the azimuth was sampled using variable steps of dφ = 360°/max{1, 36 sin(θ)} (i.e., using only one point when θ = 0° and 180° and 36 points when θ = 90°). In this way, the differential solid angle associated with a specific combination of θ and φ has a roughly constant value: dΩ = sin(θ) dθ dφ. With these settings, the total number of sampled orientations was *M* = 390.

The total energy can be converted into probability associated with the orientation, since the orientations with the lowest energy should be those most likely of occur. According to the Boltzmann equilibrium distribution^52−54^
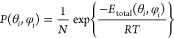
11where the normalization constant *N* is given by
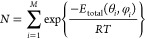
12

Note that the contributions representing different orientations can be summed evenly in eq 10, because the differential solid angles associated with them are identical. The interaction energies can be used in place of the total energies in eqs 11 and 12, leading to the same probabilities.

### Adsorption Equilibria

The probabilities of eq 11 depend on the relative energies of the adsorbed states, but they are independent of the overall adsorption energy defined by eq 10. Adding or subtracting a constant value to the total energies leads to the same probabilities, because of the normalization in eq 12. However, a change in the overall adsorption energy will have an effect on the degree of coverage of the electrode by the proteins, for a given protein concentration in solution. This can be described using Langmuir’s theory of adsorption.^52−54^ Langmuir’s theory assumes that the proteins do not interact with each other, neither in solution nor with the electrode. This is certainly an idealization, but it is consistent with our absorption calculations, which consider a single protein on the electrode.

Let χ be the overall coverage of the electrode, defined as the fraction of its area covered with proteins (0 ≤ χ ≤ 1). We also define χ(θ_*i*_, φ_*i*_) as the fraction of the electrode area covered by the proteins with a specific orientation, out of *M* possibilities. These are related by
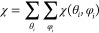
13

Clearly, χ(θ_*i*_, φ_*i*_) should be proportional to the probabilities of eq 11. We may thus write
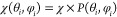
14

The Langmuir adsoption equation relates χ to the protein’s osmotic pressure Π in solution:
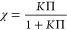
15Here *K* is an equilibrium constant for the overall protein adsorption. χ is proportional to Π (and to the protein concentration in solution, if this behaves ideally) when Π ≪ *K*^–1^, but it saturates at 1 when Π ≫ *K*^–1^. The latter situation corresponds to the formation of a protein monolayer on the electrode surface. *K* summarizes the effects of all the individual equilibria, between hydrogenases in solution (*H*_sol_) and hydrogenases on the surface (*H*_surface_):
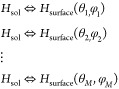
16

Each has its own equilibrium constant *K*(θ_*i*_, φ_*i*_), related as follows to the interaction free energies of eq 10:^54^
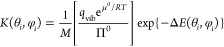
17

The square brackets collect quantities depending on the vibrational motion of the adsorbed proteins on the surface (*q*_vib_ is a vibrational partition function) and the osmotic pressure and the chemical potential of the proteins in solution, at some reference concentration (Π^0^, μ^0^). These would be difficult if not impossible to calculate. Therefore, we simply assume them to be constant (independent of protein orientation). The 1/*M* prefactor in eq 17 accounts for the loss in rotational entropy, which occurs when the protein passes from the solution state to the adsorbed one, whereby it adopts a specific orientation. The overall equilibrium constant appearing in eq 14 can be obtained as the sum of the individual, orientation-dependent constants:
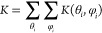
18

Note the consistency among eqs 13 and 18. All these equilibrium constants depend on pH, salinity, and electrostatic potential at the electrode.

### Electron Transfer Rates and Currents

As stated in the Introduction, the enzyme orientation on the electrode affects its catalytic efficiency,^2^ because the active site may not be equally accessible to the reactants and products (H_2_ and H^+^) and electron transport to/from the electrode may be more or less difficult. Here we concentrate on the latter, since this is often the rate-limiting step for the redox reactions.^1,22^ Modeling accurately the electron transport within a protein^55^ and between the protein and the electrode is a complex task, which is beyond the scope of this article. Instead, we adopt a simple model which is based on the notion that electron transport in hydrogenases occurs by classical hopping or quantum mechanical tunneling—the distinction is not so important in the context of this paper—between the electrode and the external iron–sulfur cluster, and from there to the active site though the other clusters.

In general, the rate of electron transfer between two sites (the electrode and the external cluster, in this case) decays exponentially with the distance *r*:^52^
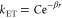
19where β determines the rate of decay and *C* absorbs all other factors affecting the electron transfer rate (e.g., free-energy difference between the electron donor and acceptor, the reorganization energy, and temperature, according to the Marcus theory of electron transfer^52,55^). In our case, each protein orientation is characterized by a different distance, so that *r* = *r*(θ_*i*_, φ_*i*_). The distance from the iron atom coordinated to histidine 187 to the surface of the electrode was measured for this purpose. We have extracted the decay constant β = 0.45 Å^–1^ from a series of values calculated by Petrenko and Stein, for a model of a similar hydrogenase on a graphene platelet (see data and plots in the Supporting Information).^56^ We point out that the value β = 1.4 Å^–1^ has been used in other publications (see, e.g., ref (57)), but this is not expected to be universal and may depend on the protein’s secondary structure features.^52^ A numerical comparison of the results from these two values is also given in the Supporting Information.

**Figure 4:**
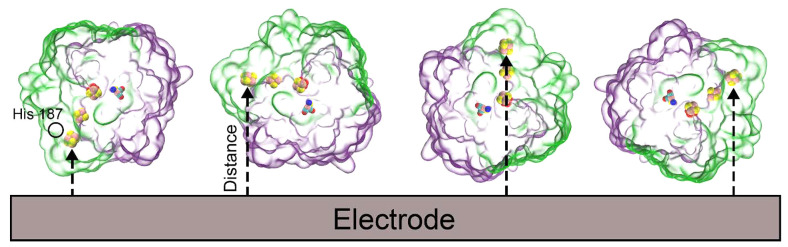
Model for calculation of the overall electric current. The external iron–sulfur cluster is the entry point for the flux of electrons to the enzyme, and its distance from the electrode has a direct impact on the rate of electron transfer.

The rate of hydrogen conversion by an individual enzyme with a specific orientation would be difficult, if not impossible, to measure. However, from a practical point of view, the most important quantity is the overall hydrogen conversion rate, or the overall electric current. As illustrated in Figure 4, the proteins adsorbed on a flat electrode may have different orientations, reflecting the probability distribution defined above. The total electric current can then be estimated by summing over all orientations, multiplying their probabilities by their respective electron transfer rates. The current should also the proportional to the area of the electrode (*A*) and to its degree of coverage (χ). We thus arrive at the expression
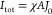
20where *J*_0_ is a reference current density, independent of protein concentration and electrode area:
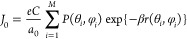
21In eq 21 we have assumed that *a*_0_, the area occupied by one adsorbed protein, is independent of its orientation. This is reasonable, considering the near-spherical shape of this hydrogenase.

The previous equations allow us to obtain the dependence of the electric current (or the hydrogen conversion rate) on the solution osmotic pressure (or the protein concentration in solution). Both the currents and the osmotic pressures can only be given in arbitrary units (AU), because of the uncertainties on the prefactors entering eqs 17 and 21. Despite this limitation, we will be able to calculate the dependence of the current on experimentally controllable variables such as the solution pH, salt concentration, and electrode potential. These prefactors will be discarded in the calculations that follow, but they may be used as adjustable parameters when fitting experimental data.

### Analysis and Postprocessing

It is often desirable to analyze the results of a calculation in the form of an image, to understand better what happens when the protein comes close to the electrode. To this purpose, we wrote a Python script to generate a Visualization Toolkit (vtk) file that could be visualized using Paraview software.^58^ A vtk file combines information on the meshes that represent surfaces of the protein and the electrode and the electrostatic potential calculated for every triangle of those meshes.

## Results and Discussion

### Minimum-Energy Orientations

**Figure 5:**
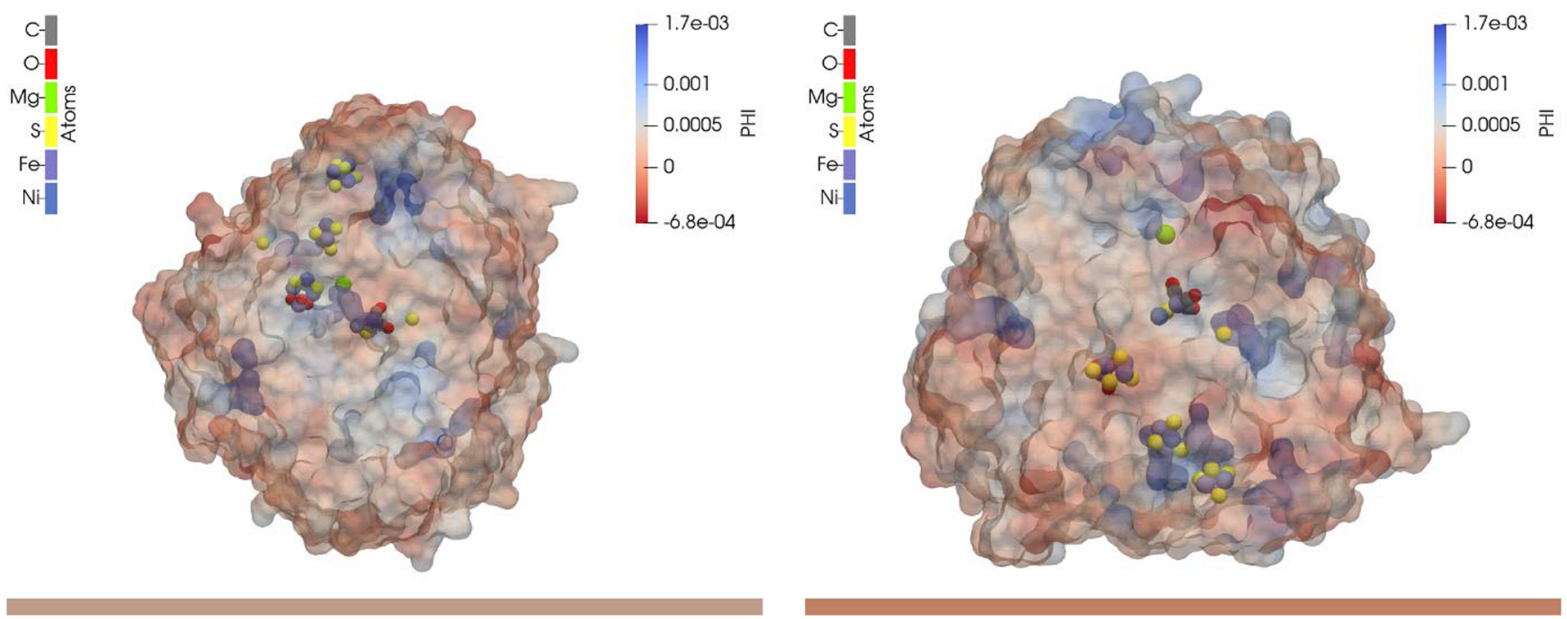
Electrostatic potentials of 1e3d hydrogenase with two different orientations. Left: θ = 40° and φ = 98°, for ϕ_e_ = 0.05 V, *I* = 0.15 M, and pH 5. Right: θ = 120° and φ = 248°, for ϕ_e_ = 0.0 V, *I* = 0.0 M, and pH 5.

Different protein orientations (390 of them, overall) were tested for each combination of experimentally controllable variables: pH, electrostatic potential on the electrode ϕ_e_, and ionic strength *I*. The orientations with the lowest interaction energies are presented in Table 2, together with their probabilities and other salient properties. According to eqs 10 and 11, the probabilities are normalized by summing over all θ and φ angles (for given pH, ϕ_e_, and *I*). Figure 5 shows plots of the calculated electrostatic potential, for two representative minimum-energy orientations.

**Table 2:** Protein Orientations (θ_min_, φ_min_) with the Lowest Energy, for Each Combination of pH, ϕ_e_, and *I*^a^

	pH	*θ*_min_(deg)	*φ*_min_(deg)	Δ*E*_min_(kJ mol^–1^)	*P*_min_	*r*_min_(Å)	AA_min_	Q10_min_ (*e*)	Qe_min_ (*e*)	*J*_0_ (AU)	*K* (AU)
*I* = 0.15 M,ϕ_e_ = −0.05 V	5	40	98	–8.15	0.031	66.82	Lys 139	0	–356.86	4.58	2.20
	6	80	131	–8.73	0.026	50.07	Phe 354	–3	–357.45	6.64	1.61
	7	80	131	–8.58	0.045	50.07	Phe 354	–3	–356.00	6.31	1.80
	8	170	60	–4.02	0.015	48.14	Phe 354	2	–360.02	5.50	0.84
	9	130	235	–9.30	0.012	16.28	Asp 197	–2	–355.39	4.81	8.81
*I* = 0.15 M,ϕ_e_ = 0.0 V	5	60	273	–6.93	0.025	17.26	Asp 197	0	–1.35	6.30	1.61
	6	60	273	–5.89	0.017	17.26	Asp 197	0	–0.56	5.51	1.59
	7	80	131	–5.52	0.016	50.07	Phe 354	–3	2.39	5.04	1.47
	8	110	270	–4.78	0.011	20.58	Ala 198	–2	0.32	5.27	1.55
	9	80	131	–5.18	0.013	50.07	Phe 354	–3	3.90	5.67	1.54
*I* = 0.15 M,ϕ_e_ = +0.05 V	5	40	98	–9.54	0.198	66.82	Lys 139	0	354.42	8.02	0.61
	6	120	261	–9.54	0.033	18.17	Lys 194	1	355.31	6.77	3.65
	7	120	261	–5.80	0.021	18.17	Lys 194	0	357.05	6.25	1.25
	8	130	180	–7.21	0.016	24.18	Asp 480	–2	361.72	5.59	2.92
	9	100	44	–13.85	0.035	51.57	Thr 12	2	359.89	6.51	19.7
*I* = 0.00 M,ϕ_e_ = 0.0 V	5	120	248	–42.86	0.156	17.13	Lys 194	4	–13.26	13.1	5.3 × 10^5^
	6	120	261	–13.12	0.061	18.17	Lys 194	1	–3.50	8.58	8.35
	7	80	131	–17.03	0.082	50.07	Phe 354	–3	5.46	4.25	30.2
	8	80	131	–36.32	0.068	50.07	Phe 354	–3	11.54	2.01	9.9 × 10^4^
	9	80	131	–53.43	0.133	50.07	Phe 354	–3	14.16	1.08	4.4 × 10^7^

a The table gives also, for these orientations, the adsorption energy (Δ*E*_min_), the probability (*P*_min_), the distance of the external iron–sulfur cluster from the electrode (*r*_min_), the closest amino acid to the electrode (AA_min_), the net charge of the 10 closest amino acids (Q10_min_), and the overall charge on the electrode (Qe_min_). The reference current densities (*J*_0_, eq 21) and the equilibrium constant for adsorption (*K*, eq 18) depend on all possible orientations.

The interaction energies are negative in all cases, indicating that the adsorbed state is stable with respect to the one with the protein away from the electrode. On average, the ionic strength appears to be the most important variable determining the strength of absorption, measured by |Δ*E*_min_|. This is mostly lower than 10 kJ mol^–1^ in the presence of salt (with only one exception, for ϕ_e_ = +0.05 V at pH 9), but it ranges between 13 and 53 kJ mol^–1^ in the salt-free cases. This is understandable, due to the screening effect of the dissolved ions on electrostatic interactions. Many experiments have corroborated the hypothesis that the amount of adsorbed proteins on charged surfaces decreases as the ionic strength increases.^27,59−63^ An enhanced adsorption is in principle an advantage for electrode functionalization. We point out that it might not be feasible in practice to work under salt-free or salt-poor conditions, as they would reduce the electrical conductivity of the solution and possibly also the stability of the enzyme. However, the salt-free case is still interesting from a fundamental perspective, as it sets an upper limit to the strength and to the spatial range of the electrostatic interactions that can be expected in these systems.

Boubeta et al.^24^ studied the interaction of lysozyme and other proteins with a negatively charged surface. They suggest that when electrostatic interactions are the main factor that determines the adsorption of a protein, two mechanisms can be considered to describe them. The first one is charge regulation (CR), whereby the protonation state of an amino acid depends not only on the pH of the solution but also on the chemical environment on the amino acid, the presence of charged groups, and the local dielectric properties.^64,65^ Placing the protein near a highly charged surface will modify the p*K*_a_ of the amino acids close to it.^66,67^ The second mechanism is charge patches (CP), whereby the protein tends to maximize the number of oppositely charged amino acids next to the surface.

Concerning the role of the electrode’s potential ϕ_e_, one might expect that the adsorption of the protein is favored when the sign of the potential is opposite that of the protein’s net charge. However, a number of studies have shown that proteins can adsorb on modified surfaces even when the charges of the protein and the surface have the same sign, possibly due to a high local concentration of amino acids with the opposite charge (i.e., by a CP-type mechanism).^59−61,68,69^

Indeed, we find that the electrostatic potential on the electrode affects the minimum-energy orientation of the protein, but it does not produce a large change in the overall absorption energy. In many cases, the changes are not easy to interpret. One situation where the changes appear to be systematic is at pH 8. Here the (θ_min_, φ_min_) angles change when ϕ_e_ changes from −0.05 to +0.05 V, indicating a reorientation of the protein due to the potential. However, |Δ*E*_min_| changes by a relatively small amount, from 5 to 7 kJ mol^–1^. This is at first surprising, considering that the protein has a net change of −12.4*e* (see Table 1), and the overall charge on the electrode changes from −360*e* to +361*e*. However, one should also remember that the charge on the electrode is actually distributed, being roughly proportional to its size. Hence, a large electrode implies a large overall charge, but this does not automatically translate into a strong local interaction.

It is interesting to compare the total charge on the electrode when ϕ_e_ = 0.00 V, as a function of pH and ionic strength. In this case, the total charge is zero when the protein is at infinite distance from the electrode. When the protein approaches the electrode and adsorbs on it, it induces a negative charge when it is positive (at acidic pH, see Table 1) and a positive charge when it is negative (at basic pH, see Table 1). This effect is strongest in the salt-free case, so that the induced charge on the electrode compensates almost exactly the total charge of the protein (about ±15 at pH 5 and 8), giving an electrically neutral electrode–protein complex.

Our calculations do not include the CR mechanism, because the protein charges are assumed to be independent of its orientation and distance from the electrode. However, we may consider the possible role of the CP mechanism. To do so, we have identified the amino acid closest to the surface for each orientation and calculated the total charge on the “patch” formed by the 10 closest amino acids. These data are also given in Table 2. One recurring orientation is that with θ = 80° and φ = 131°, in which the amino acid closest to the surface is Phe 354, which is electrically neutral and nonpolar. However, if we consider the whole patch, we find a value of −3*e*, at pH ≥6. This explains the recurrence of this particular orientation in Table 2 (7 instances out of 20). There is only one other case with a larger charge of the patch (+4*e*), at pH 5 in pure water. In this case the closest amino acid is a positively charged Lys 194, and the absorption energy is relatively large (−42 kJ/mol). Interestingly, this is the closest amino acid also at pH 6, with a slightly different orientation. However, now the overall charge of the patch is only +1 (that of Lys itself), and consequently the value of the absorption energy is significantly smaller (−13 kJ/mol). Note that the presence of the Lys residue close to the electrode occurs also in other entries of Table 2, indicating that it may be crucial for achieving strong absorption on the electrode.

The probabilities of the minimum-energy orientations (*P*_min_ in Table 2) are always relatively low, typically less than 0.1. This indicates that this orientation never dominates the distributions of absorption angles, but there is instead an equilibrium involving several other orientations that are only slightly higher in energy. There are only three entries in Table 2 where *P*_min_ > 0.1. Two of them occur in the salt-free cases, where a large charge of the patch favors a very strong adsorption. The third case occurs in the saline solution at pH 5, with a positive electrode potential. The net charge of the patch is zero, but interestingly the amino acid closest to the surface is again a Lys.

With respect to the effect of the pH, many studies report that protein adsorption is maximum at pHs near the isoelectric point (IEP) of the protein.^60,70,71^ Some of these observations could be ascribed to the role of nonelectrostatic forces (e.g., van der Waals and hydrophobic interactions), which may lead to protein adsorption even in the presence of unfavorable electrostatic interactions.^27,57,72^ These nonelectrostatic forces could be included in an approximate way by a solvent-accessible-surface-area (SASA) model.^73^ In view of the near-spherical nature of our hydrogenase, the surface area is independent of orientation, unless the interaction is so strong as to deform and disrupt the proteins’ three-dimensional structures (possible but unlikely under many practical circumstances, as this would impair their catalytic activities). Thus, an overall enhancement or reduction of the protein–electrode interaction would leave the equilibrium distribution essentially unchanged. We also point out that the potential at the electrode is zero or relatively low in our calculations (±0.05 V). According to some reports, strong electric fields produced by applying higher voltages could change the preferential orientation of the protein and possibly even disrupt it, either by physical denaturation or by chemical reaction with radicals formed at the electrodes.^21,74−77^ These phenomena are clearly crucial for the operation of enzyme-based electrodes, but their investigation is beyond the possibilities of the present computational approach.

### Orientational Distributions

We now turn to discuss the consequences of the enzyme orientation on its catalytic efficiency. According to our previous discussion, this is expected to depend on the rate of electron transfer from the electrode to the external iron–sulfur cluster. Thus, the two orientations shown in Figure 5 can be expected to show different activities, since the external iron–sulfur cluster is far from the electrode on the left-hand side and very close to it on the right-hand side. The distances are reported in Table 2, under the *r*_min_ column. The shortest distance of all is 16.28 Å, obtained in a salt solution at pH 9 with a negative potential (θ = 130° and φ = 235°).

However, specific orientations are not representative of the whole system, because they account for a relatively small fraction of all the adsorbed proteins. Those in Figure 5 have probabilities of *P*_min_ = 0.198 and 0.156, respectively, while the third one discussed above has *P*_min_ = 0.012 (see again Table 2). In all cases, more than 80% of the proteins would adopt an arrangement different from the minimum. There is a thermal and dynamical equilibrium between a multitude of orientations, each with a different catalytic activity.

**Figure 6:**
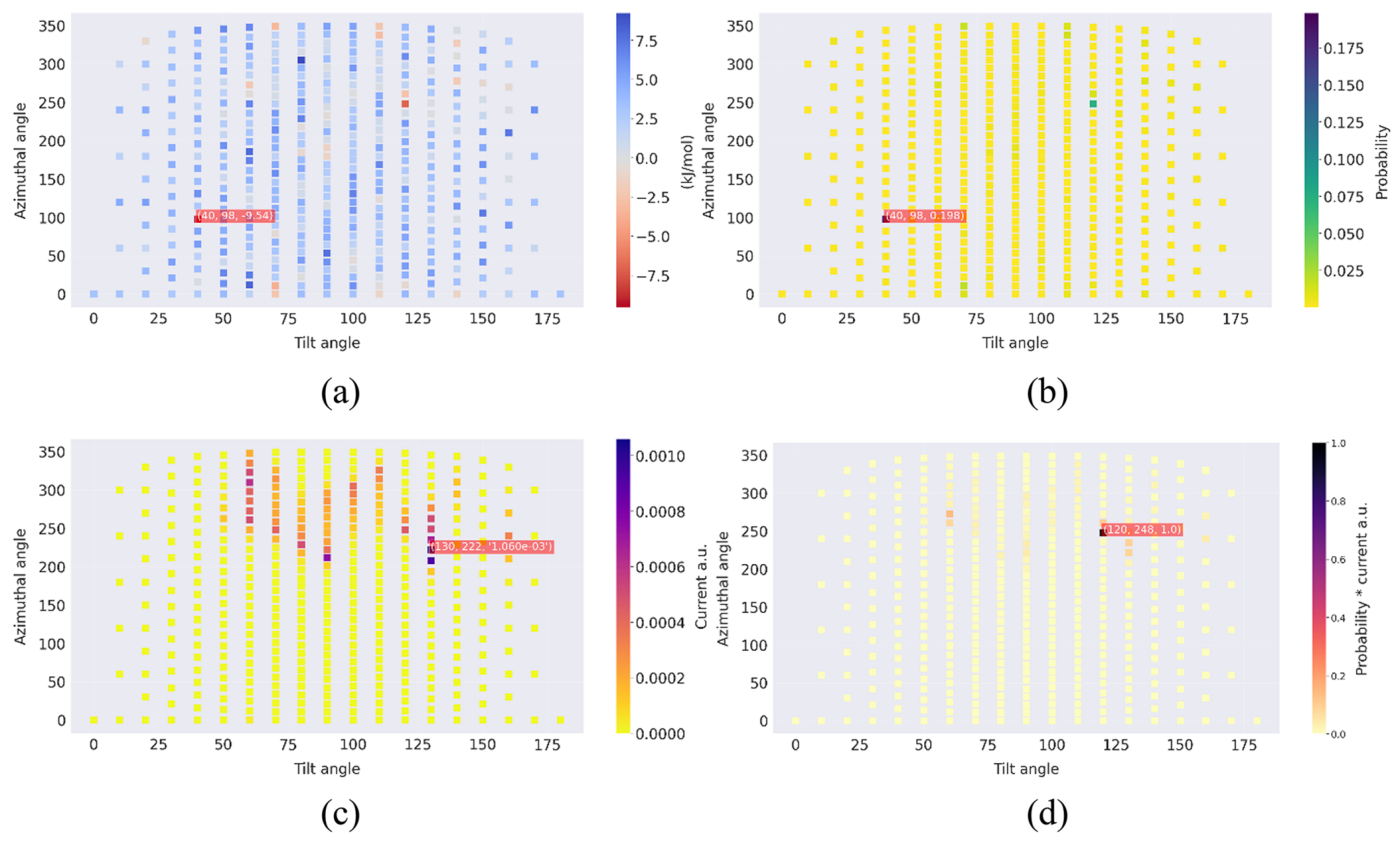
Interaction of 1e3d hydrogenase with the electrode at ϕ_e_ = 0.05 V, in a solution with *I* = 0.15 M and pH 5. (a) Heatmap plot of interaction energies Δ*E* (kJ mol^–1^). (b) Boltzmann probabilities. (c) Electron transfer rates (AU). (d) Product of Boltzmann probability and electron transfer rates (AU).

**Figure 7:**
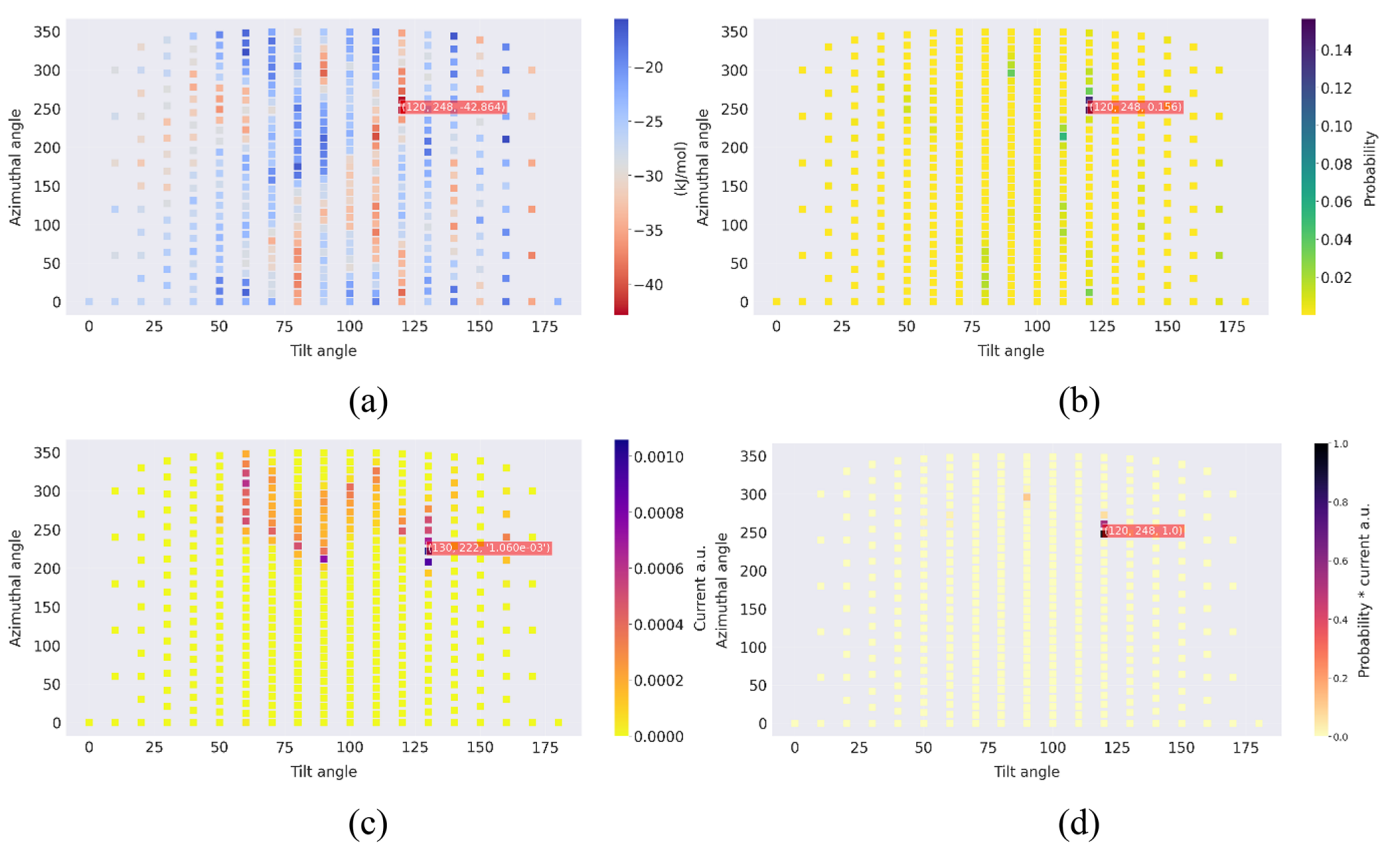
Interaction of 1e3d hydrogenase with the electrode at ϕ_e_ = 0.0 V, in a solution with *I* = 0.0 M and pH 5. Panels a–d as in Figure 6.

Figures 6 and 7 illustrate the absorption energies (panel a), the Boltzmann probabilities (panel b), and the electron transfer rates (panel c), as two-dimensional heatmaps as a function of the tilt (θ) and azimuthal (φ) angles. Note that Figures 6c and 7c are identical since, in the approximation of eq 19, the electron transfer rates depend only on the electrode–cluster distance. Figures 6d and 7d display the product of the probabilities and currents, as this is the quantity that determines the average contribution of each orientation to the current density, according to eq 21. Clearly, a high current density is obtained only when there is a good “overlap” between the Boltzmann probabilities (panel b) and the electron transfer rates (panel c).

The final results for the reference current densities (*J*_0_) are reported in the last-but-one column of Table 2. Despite the large differences in the protein orientations at the minima, and in the electron transfer rates at these minima, the overall currents are remarkably similar. The highest and the lowest values of the current density, occurring in the salt-free solutions, differ by only 1 order of magnitude. If we consider only the salt-containing solutions, the differences are even lower.

### Absorption Equilibria and Currents

As a final step, we consider the effect of protein concentration or, equivalently, its osmotic pressure (Π). According to the Langmuir model, this affects the electrode coverage (χ) through the overall equilibrium constant *K* (see eq 15). The value of *K* may be dominated by a few strongly adsorbed orientations, or it may result from several, roughly equivalent ones.

**Figure 8:**
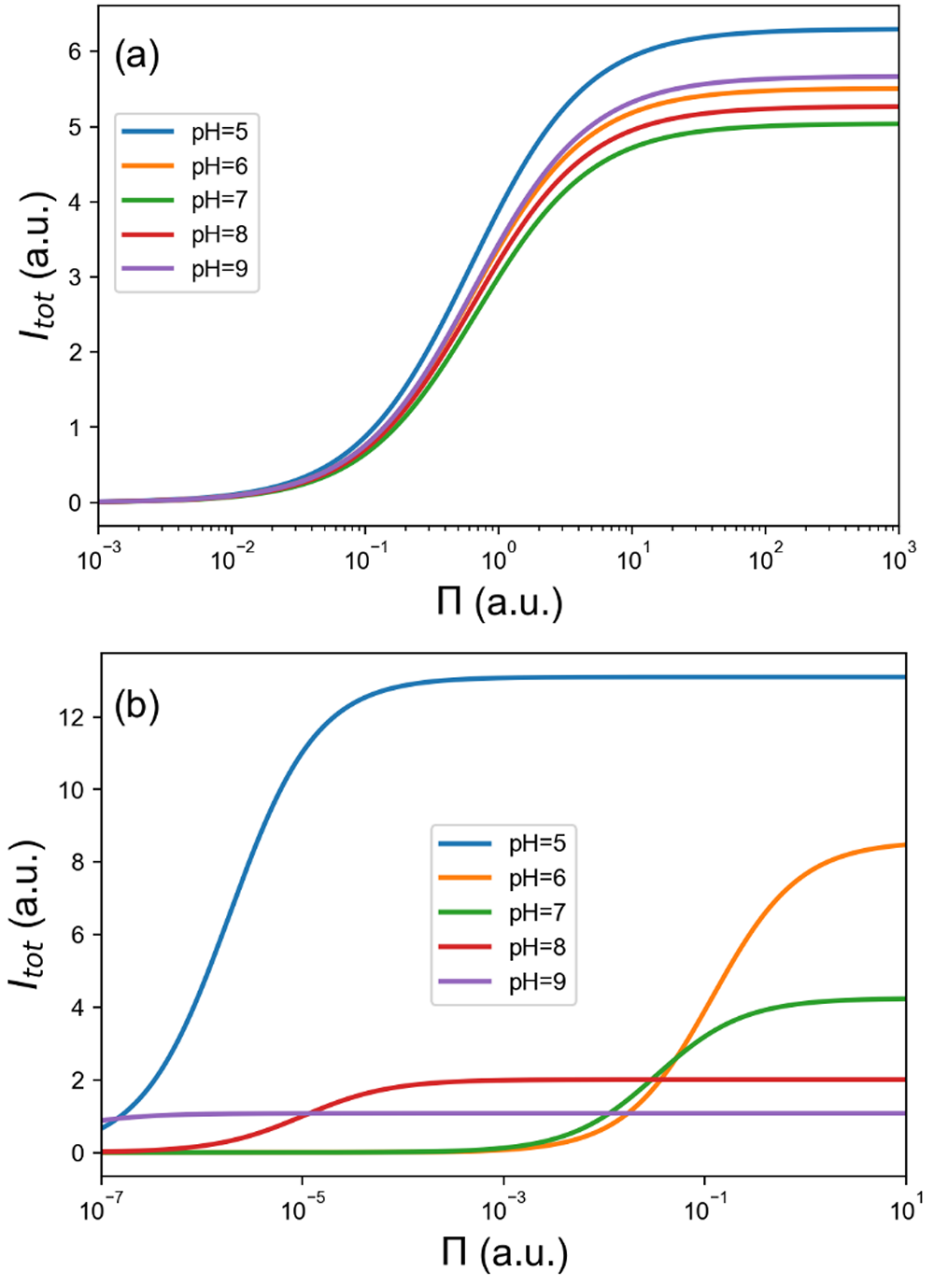
Dependence of current on pH and protein osmotic pressure, at zero electrode potential. (a) Salty solutions, *I* = 0.15 M; (b) salt-free solutions, *I* = 0.00 M.

Figure 8 illustrates the behavior of the total currents at zero electrode potential in salty and salt-free solutions, respectively (panels a and b). In the former, there is little difference between the results at different pHs, confirming our earlier conclusions. The solution at pH 5 seems to be marginally better, because of the combination of a slightly higher *J*_0_ (determining the saturation value of the current at large protein concentrations) and a slightly higher K (determining the position of the inflection point in the Langmuir isotherms). There is a much greater dependence on pH in the salt-free solutions. The cases at pH 8 and 9 are characterized by strong absorption (large *K*), but the enzyme is essentially locked in an unfavorable orientation for electron transfer (small *J*_0_). At pH 6 and 7 we have the opposite condition. By far, the best situation occurs again at pH 5, thanks to a favorable combination of large *K* and large *J*_0_.

**Figure 9:**
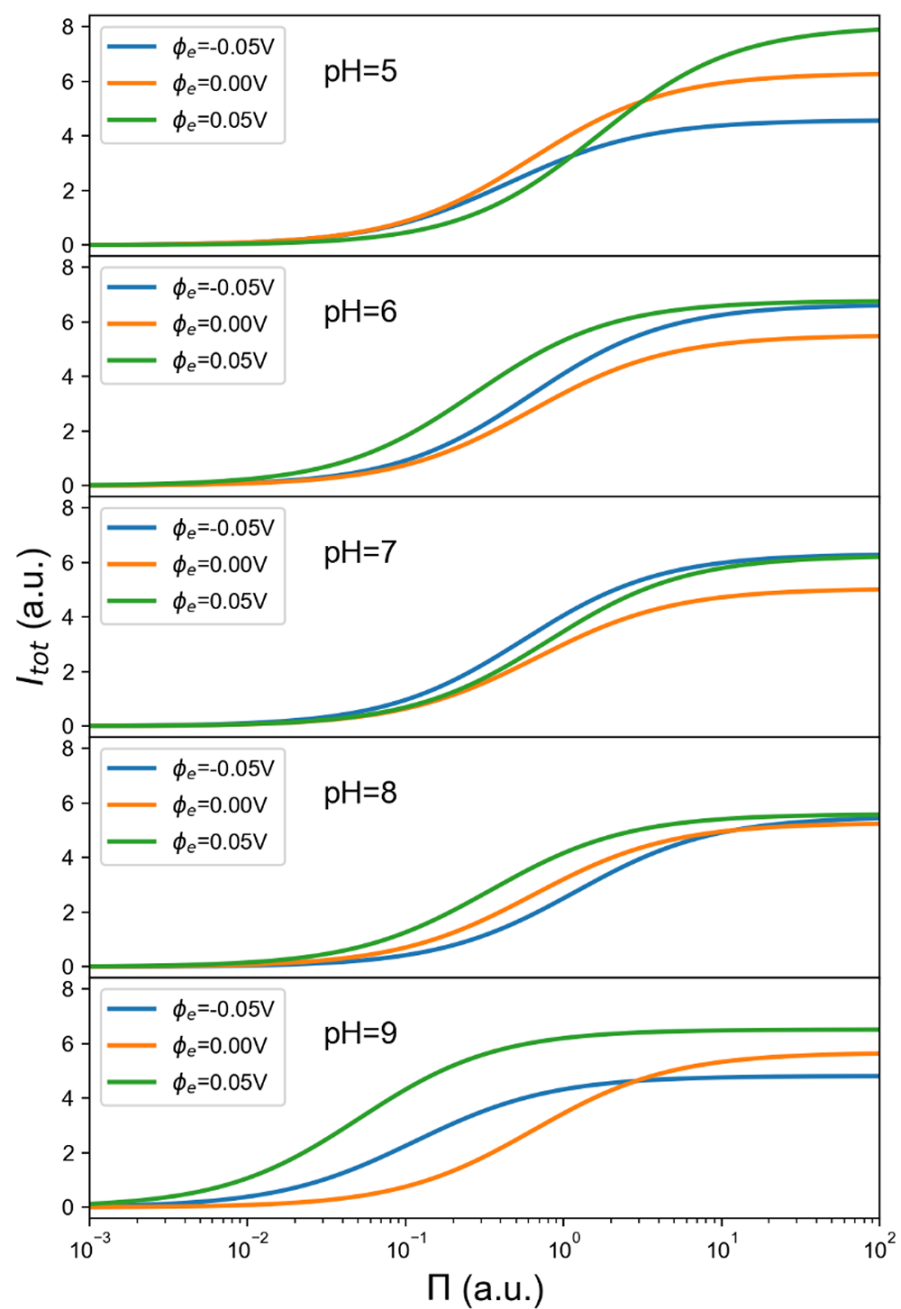
Effects of electrode potential, protein osmotic pressure, and pH on total current, for saline solutions with *I* = 0.15 M.

Figure 9 demonstrates the effect of modulating the electrode potential, within the range compatible with the applicability of the linearized Poisson–Boltzmann equation (−0.05 V ≤ ϕ_e_ ≤ 0.05 V). The most visible changes occur at the two ends of the pH spectrum, where the protein has either a large and positive charge or a large and negative charge. A negatively charged protein (pH 9) has a much greater affinity for the electrode at positive potential. Interestingly, in order of decreasing affinity, the electrode at negative potential comes before the electrode at zero potential. This is counterintuitive and could not have been expected on the basis of the protein’s charge and dipole. Considering the positively charged protein (pH 5), absorption is more favorable with a negative potential, but the sensitivity of the equilibrium constant to this variable is much lower in this case. There is however a greater sensitivity of the reference current density, which can be ascribed to a significant change in the distribution of the protein orientations. Note that the moduli of protein charge and dipole moment are similar in these two cases (see again Table 1). Again, we believe that this effect of the electrode potential could not have been easily predicted, on the basis of these descriptors of the protein charge distributions.

As a final remark, we point out again that the Langmuir model neglects protein–protein interactions and does account for the possible formation of multilayer structures. These have been observed for a number of proteins, especially at high applied voltages (of the order of ±1 V), depending also on their degree of conformational rigidity.^78^

## Conclusions

We have presented the results of Poisson–Boltzmann calculations for the adsorption of a hydrogenase on a planar conducting electrode. One of the main goals was to determine the possibility to control the orientation of the protein, exploiting electrostatic interactions. These are not the only ones, but they are expected to dominate at least in the absence of a specific functionalization of the electrode (e.g., to produce a covalent attachment). According to our calculations, the adsorption of the enzyme on the electrode depends on the solution’s pH and salinity and the electrostatic potential at the electrode. We have found that the latter does not affect much the adsorption energy of the protein, but its sign has a clear effect on the orientation. The strongest orientations are obtained in the presence of charge patches on the protein’s surface, whose presence depends on the solution pH. The overall interaction is largest in salt-free solutions, where electrostatic interactions are not screened by the presence of counterions.

The total current, which measures the overall rate of the redox reactions at the active sites of the adsorbed enzymes, is maximum when there is a good match between the Boltzmann probabilities of the orientations, their electron transfer rates, and the strength of the protein absorption on the electrode surface. In general, we do not find situations dominated by a single protein orientation, but the overall current depends on a whole population of absorbed states. However, we have observed that a number minimum-energy states are characterized by a Lys residue close to the electrode’s surface. It would be interesting to further pursue and check this observation, by studying other hydrogenases or by introducing further Lys residues on the protein’s exterior, at favorable positions with respect to the external [3Fe4S] cluster.

Despite a number of approximations, we believe that the present computational approach has demonstrated its usefulness, first and foremost because of the physical insights can it can generate. We are now applying it to other hydrogenases, starting from the thermal- and oxygen-tolerant ones that are more interesting for renewable-energy applications. On a parallel line, we are interested in extending it by seeking the nonlinear solutions of the full Poisson–Boltzmann equation, including also charge regulation effects, larger values of the electrode potential, and hydrophobic effects by a SASA model. We point out that SASA models are widely used for protein solvation, but their applicability to electrode surfaces cannot be taken for granted. The configurations generated by these calculations would be useful also as a starting point for molecular dynamics simulations, accounting for more specific nonelectrostatic interactions and for the proteins’ flexibility. Indeed, some flexibility is known to be essential for the functionality of an enzyme, for example, to assist the diffusion of the substrates to and away from the catalytic site. The molecular dynamics results could also be used the other way round, as an input to further Poisson–Boltzmann-based analyses of electrostatic effects and free energies^73^ and to the evaluation of electron transfer rates.^56,79^ Over a longer time scale, the enzymes’ reorientational motion, which can be induced for example by a large switch in the electrode’s voltage,^57,80,81^ could also be treated by a coupling a nonlinear Poisson–Boltzmann solver with a microhydrodynamic description of the surrounding fluid.^82^ Of course, such refinements of the model should be stimulated and cross-checked by experiments, including ones based on modern in situ operando spectroscopies that provide information about local interactions at the protein–electrode interface.^83,84^

## Supporting Information Available

Additional plots and data on electron transfer between protein and electrode; summary of ORCA calculations for the clusters; customized Amber force field file for pdb2prq; example input files for Nanoshaper and PyGBe; Python script to generate vtk files from PyGBe outputs, for visualization (ZIP)

## References

[ref1] Lubitz W.; Ogata H.; Rüdiger O.; Reijerse E. Hydrogenases. Chem. Rev. 2014, 114 (8), 4081–4148. 10.1021/cr4005814. 24655035

[ref2] Lojou E. Hydrogenases as Catalysts for Fuel Cells: Strategies for Efficient Immobilization at Electrode Interfaces. Electrochim. Acta 2011, 56 (28), 10385–10397. 10.1016/j.electacta.2011.03.002.

[ref3] Vincent K. A.; Parkin A.; Armstrong F. A. Investigating and Exploiting the Electrocatalytic Properties of Hydrogenases. Chem. Rev. 2007, 107 (10), 4366–4413. 10.1021/cr050191u. 17845060

[ref4] Luo X.; Brugna M.; Tron-Infossi P.; Giudici-Orticoni M. T.; Lojou É. Immobilization of the Hyperthermophilic Hydrogenase from Aquifex Aeolicus Bacterium onto Gold and Carbon Nanotube Electrodes for Efficient H2 Oxidation. JBIC Journal of Biological Inorganic Chemistry 2009, 14 (8), 1275–1288. 10.1007/s00775-009-0572-y. 19629542

[ref5] Jugder B.-E.; Welch J.; Aguey-Zinsou K.-F.; Marquis C. P. Fundamentals and Electrochemical Applications of [Ni–Fe]-Uptake Hydrogenases. RSC Adv. 2013, 3 (22), 8142 10.1039/c3ra22668a.

[ref6] Breglia R.; Ruiz-Rodriguez M. A.; Vitriolo A.; Gonzàlez-Laredo R. F.; de Gioia L.; Greco C.; Bruschi M. Theoretical Insights into [NiFe]-Hydrogenases Oxidation Resulting in a Slowly Reactivating Inactive State. JBIC Journal of Biological Inorganic Chemistry 2017, 22 (1), 137–151. 10.1007/s00775-016-1416-1. 27873068

[ref7] Siegbahn P. E. M.; Tye J. W.; Hall M. B. Computational Studies of [NiFe] and [FeFe] Hydrogenases. Chem. Rev. 2007, 107 (10), 4414–4435. 10.1021/cr050185y. 17927160

[ref8] Karyakin A. A.; Morozov S. V.; Karyakina E. E.; Zorin N. A.; Perelygin V. V.; Cosnier S. Hydrogenase Electrodes for Fuel Cells. Biochem. Soc. Trans. 2005, 33 (1), 73–75. 10.1042/BST0330073. 15667269

[ref9] Lee C.; Park H. S.; Fontecilla-Camps J. C.; Reisner E. Photoelectrochemical H _2_ Evolution with a Hydrogenase Immobilized on a TiO _2_ -Protected Silicon Electrode. Angew. Chem. 2016, 128 (20), 6075–6078. 10.1002/ange.201511822. PMC4982046 27570301

[ref10] Xiao X.; Xia H.; Wu R.; Bai L.; Yan L.; Magner E.; Cosnier S.; Lojou E.; Zhu Z.; Liu A. Tackling the Challenges of Enzymatic (Bio)Fuel Cells. Chem. Rev. 2019, 119 (16), 9509–9558. 10.1021/acs.chemrev.9b00115. 31243999

[ref11] Mersch D.; Lee C.-Y.; Zhang J. Z.; Brinkert K.; Fontecilla-Camps J. C.; Rutherford A. W.; Reisner E. Wiring of Photosystem II to Hydrogenase for Photoelectrochemical Water Splitting. J. Am. Chem. Soc. 2015, 137 (26), 8541–8549. 10.1021/jacs.5b03737. 26046591

[ref12] Morra S.; Valetti F.; Sadeghi S. J.; King P. W.; Meyer T.; Gilardi G. Direct Electrochemistry of an [FeFe]-Hydrogenase on a TiO2 Electrode. Chem. Commun. 2011, 47 (38), 10566 10.1039/c1cc14535e. 21863186

[ref13] Monsalve K.; Roger M.; Gutierrez-Sanchez C.; Ilbert M.; Nitsche S.; Byrne-Kodjabachian D.; Marchi V.; Lojou E. Hydrogen Bioelectrooxidation on Gold Nanoparticle-Based Electrodes Modified by Aquifex Aeolicus Hydrogenase: Application to Hydrogen/Oxygen Enzymatic Biofuel Cells. Bioelectrochemistry 2015, 106, 47–55. 10.1016/j.bioelechem.2015.04.010. 25960259

[ref14] Rüdiger O.; Gutiérrez-Sánchez C.; Olea D.; Pereira I. A. C.; Vélez M.; Fernández V. M.; De Lacey A. L. Enzymatic Anodes for Hydrogen Fuel Cells Based on Covalent Attachment of Ni-Fe Hydrogenases and Direct Electron Transfer to SAM-Modified Gold Electrodes. Electroanalysis 2010, 22 (7–8), 776–783. 10.1002/elan.200880002.

[ref15] Gutiérrez-Sanz Ó.; Natale P.; Márquez I.; Marques M. C.; Zacarias S.; Pita M.; Pereira I. A. C.; López-Montero I.; De Lacey A. L.; Vélez M. H _2_ -Fueled ATP Synthesis on an Electrode: Mimicking Cellular Respiration. Angew. Chem. 2016, 128 (21), 6324–6328. 10.1002/ange.201600752. PMC5132028 26991333

[ref16] Quinson J.; Hidalgo R.; Ash P. A.; Dillon F.; Grobert N.; Vincent K. A. Comparison of Carbon Materials as Electrodes for Enzyme Electrocatalysis: Hydrogenase as a Case Study. Faraday Discuss. 2014, 172, 473–496. 10.1039/C4FD00058G. 25426610

[ref17] Liu J.; Wu W.-J.; Fang F.; Zorin N. A.; Chen M.; Qian D.-J. Immobilization of Hydrogenase on Carbon Nanotube Polyelectrolytes as Heterogeneous Catalysts for Electrocatalytic Interconversion of Protons and Hydrogen. J. Nanopart. Res. 2016, 18 (8), 220 10.1007/s11051-016-3530-y.

[ref18] Mazurenko I.; Monsalve K.; Infossi P.; Giudici-Orticoni M.-T.; Topin F.; Mano N.; Lojou E. Impact of Substrate Diffusion and Enzyme Distribution in 3D-Porous Electrodes: A Combined Electrochemical and Modelling Study of a Thermostable H_2_/O_2_ Enzymatic Fuel Cell. Energy Environ. Sci. 2017, 10 (9), 1966–1982. 10.1039/C7EE01830D.

[ref19] Rüdiger O.; Abad J. M.; Hatchikian E. C.; Fernandez V. M.; de Lacey A. L. Oriented Immobilization of *Desulfovibrio Gi Gas* Hydrogenase onto Carbon Electrodes by Covalent Bonds for Nonmediated Oxidation of H_2_. J. Am. Chem. Soc. 2005, 127 (46), 16008–16009. 10.1021/ja0554312. 16287271

[ref20] Gutiérrez-Sánchez C.; Olea D.; Marques M.; Fernández V. M.; Pereira I. A. C.; Vélez M.; de Lacey A. L. Oriented Immobilization of a Membrane-Bound Hydrogenase onto an Electrode for Direct Electron Transfer. Langmuir 2011, 27 (10), 6449–6457. 10.1021/la200141t. 21491850

[ref21] Heidary N.; Utesch T.; Zerball M.; Horch M.; Millo D.; Fritsch J.; Lenz O.; von Klitzing R.; Hildebrandt P.; Fischer A.; Mroginski M. A.; Zebger I. Orientation-Controlled Electrocatalytic Efficiency of an Adsorbed Oxygen-Tolerant Hydrogenase. PLoS One 2015, 10 (11), e0143101 10.1371/journal.pone.0143101. 26580976 PMC4651547

[ref22] Hitaishi V.; Clement R.; Bourassin N.; Baaden M.; de Poulpiquet A.; Sacquin-Mora S.; Ciaccafava A.; Lojou E. Controlling Redox Enzyme Orientation at Planar Electrodes. Catalysts 2018, 8 (5), 192 10.3390/catal8050192.

[ref23] Oteri F.; Ciaccafava A.; Poulpiquet A. de; Baaden M.; Lojou E.; Sacquin-Mora S. The Weak, Fluctuating, Dipole Moment of Membrane-Bound Hydrogenase from Aquifex Aeolicus Accounts for Its Adaptability to Charged Electrodes. Phys. Chem. Chem. Phys. 2014, 16 (23), 11318–11322. 10.1039/C4CP00510D. 24789038

[ref24] Boubeta F. M.; Soler-Illia G. J. A. A.; Tagliazucchi M. Electrostatically Driven Protein Adsorption: Charge Patches versus Charge Regulation. Langmuir 2018, 34 (51), 15727–15738. 10.1021/acs.langmuir.8b03411. 30451508

[ref25] Tsori Y. Bistable Colloidal Orientation in Polar Liquid near a Charged Wall. J. Colloid Interface Sci. 2020, 559, 45–50. 10.1016/j.jcis.2019.09.096. 31610304

[ref26] Urzúa S. A.; Sauceda-Oloño P. Y.; García C. D.; Cooper C. D. Predicting the Orientation of Adsorbed Proteins Steered with Electric Fields Using a Simple Electrostatic Model. J. Phys. Chem. B 2022, 126 (28), 5231–5240. 10.1021/acs.jpcb.2c03118. 35819287

[ref27] Norde W. My Voyage of Discovery to Proteins in Flatland··· and Beyond. Colloids Surfaces B Biointerfaces 2008, 61 (1), 1–9. 10.1016/j.colsurfb.2007.09.029. 18023976

[ref28] Mazurenko I.; Monsalve K.; Rouhana J.; Parent P.; Laffon C.; Goff A. L.; Szunerits S.; Boukherroub R.; Giudici-Orticoni M. T.; Mano N.; Lojou E. How the Intricate Interactions between Carbon Nanotubes and Two Bilirubin Oxidases Control Direct and Mediated O2 Reduction. ACS Appl. Mater. Interfaces 2016, 8 (35), 23074–23085. 10.1021/acsami.6b07355. 27533778

[ref29] Ren P.; Chun J.; Thomas D. G.; Schnieders M. J.; Marucho M.; Zhang J.; Baker N. A. Biomolecular Electrostatics and Solvation: A Computational Perspective. Q. Rev. Biophys. 2012, 45 (4), 427–491. 10.1017/S003358351200011X. 23217364 PMC3533255

[ref30] Honig B.; Nicholls A. Classical Electrostatics in Biology and Chemistry. Science (80-.) 1995, 268 (5214), 1144–1149. 10.1126/science.7761829. 7761829

[ref31] Fogolari F.; Brigo A.; Molinari H. The Poisson-Boltzmann Equation for Biomolecular Electrostatics: A Tool for Structural Biology. Journal of Molecular Recognition 2002, 15 (6), 377–392. 10.1002/jmr.577. 12501158

[ref32] Baker N. A.; Sept D.; Joseph S.; Holst M. J.; McCammon J. A. Electrostatics of Nanosystems: Application to Microtubules and the Ribosome. Proc. Natl. Acad. Sci. U. S. A. 2001, 98 (18), 10037–10041. 10.1073/pnas.181342398. 11517324 PMC56910

[ref33] Gilson M. K.; Sharp K. A.; Honig B. H. Calculating the Electrostatic Potential of Molecules in Solution: Method and Error Assessment. J. Comput. Chem. 1988, 9 (4), 327–335. 10.1002/jcc.540090407.

[ref34] Rocchia W.; Alexov E.; Honig B. Extending the Applicability of the Nonlinear Poisson-Boltzmann Equation: Multiple Dielectric Constants and Multivalent Ions. J. Phys. Chem. B 2001, 105 (28), 6507–6514. 10.1021/jp010454y.

[ref35] Bashford D. An Object-Oriented Programming Suite for Electrostatic Effects in Biological Molecules An Experience Report on the MEAD Project. Lecture Notes in Computer Science 1997, 1343, 233–240. 10.1007/3-540-63827-X_66.

[ref36] Geng W.; Yu S.; Wei G. Treatment of Charge Singularities in Implicit Solvent Models. J. Chem. Phys. 2007, 127 (11), 114106 10.1063/1.2768064. 17887827

[ref37] Lu B.; Cheng X.; Huang J.; McCammon J. A. Order N Algorithm for Computation of Electrostatic Interactions in Biomolecular Systems. Proc. Natl. Acad. Sci. U. S. A. 2006, 103 (51), 19314–19319. 10.1073/pnas.0605166103. 17148613 PMC1748223

[ref38] Geng W.; Krasny R. A Treecode-Accelerated Boundary Integral Poisson–Boltzmann Solver for Electrostatics of Solvated Biomolecules. J. Comput. Phys. 2013, 247, 62–78. 10.1016/j.jcp.2013.03.056.

[ref39] Cooper C. D.; Barba L. A. Poisson–Boltzmann Model for Protein–Surface Electrostatic Interactions and Grid-Convergence Study Using the PyGBe Code. Comput. Phys. Commun. 2016, 202, 23–32. 10.1016/j.cpc.2015.12.019.

[ref40] Cooper C. D.; Bardhan J. P.; Barba L. A. A Biomolecular Electrostatics Solver Using Python, GPUs and Boundary Elements That Can Handle Solvent-Filled Cavities and Stern Layers. Comput. Phys. Commun. 2014, 185 (3), 720–729. 10.1016/j.cpc.2013.10.028. 25284826 PMC4179212

[ref41] Cooper C. D.; Clementi N. C.; Barba L. A. Probing Protein Orientation near Charged Nanosurfaces for Simulation-Assisted Biosensor Design. J. Chem. Phys. 2015, 143 (12), 124709 10.1063/1.4931113. 26429034

[ref42] Matias P. M.; Soares C. M.; Saraiva L. M.; Coelho R.; Morais J.; le Gall J.; Carrondo M. A. [NiFe] Hydrogenase from Desulfovibrio Desulfuricans ATCC 27774: Gene Sequencing, Three-Dimensional Structure Determination and Refinement at 1.8 Å and Modelling Studies of Its Interaction with the Tetrahaem Cytochrome c 3. JBIC Journal of Biological Inorganic Chemistry 2001, 6 (1), 63–81. 10.1007/s007750000167. 11191224

[ref43] Lee J.; Kim S.-H. *PDB Editor* : A User-Friendly Java-Based Protein Data Bank File Editor with a GUI. Acta Crystallographica Section D Biological Crystallography 2009, 65 (4), 399–402. 10.1107/S090744490900451X. 19307724

[ref44] Boettcher S. W.; Oener S. Z.; Lonergan M. C.; Surendranath Y.; Ardo S.; Brozek C.; Kempler P. A. Potentially Confusing: Potentials in Electrochemistry. ACS Energy Lett. 2021, 6, 261–266. 10.1021/acsenergylett.0c02443.

[ref45] Dolinsky T. J.; Nielsen J. E.; McCammon J. A.; Baker N. A. PDB2PQR: An Automated Pipeline for the Setup of Poisson-Boltzmann Electrostatics Calculations. Nucleic Acids Res. 2004, 32, W665–W667. 10.1093/nar/gkh381. 15215472 PMC441519

[ref46] Hornak V.; Abel R.; Okur A.; Strockbine B.; Roitberg A.; Simmerling C. Comparison of Multiple Amber Force Fields and Development of Improved Protein Backbone Parameters. Proteins: Struct., Funct., Bioinf. 2006, 65 (3), 712–725. 10.1002/prot.21123. PMC4805110 16981200

[ref47] Neese F. The ORCA Program System. WIREs Computational Molecular Science 2012, 2 (1), 73–78. 10.1002/wcms.81.

[ref48] Neese F. Software Update: The ORCA Program System, Version 4.0. WIREs Comput. Mol. Sci. 2018, 8 (1), e1327 10.1002/wcms.1327.

[ref49] Weigend F.; Ahlrichs R. Balanced Basis Sets of Split Valence, Triple Zeta Valence and Quadruple Zeta Valence Quality for H to Rn: Design and Assessment of Accuracy. Phys. Chem. Chem. Phys. 2005, 7 (18), 3297 10.1039/b508541a. 16240044

[ref50] Adamo C.; Barone V. Toward Reliable Density Functional Methods without Adjustable Parameters: The PBE0Model. J. Chem. Phys. 1999, 110 (13), 6158–6170. 10.1063/1.478522.

[ref51] Decherchi S.; Rocchia W. A General and Robust Ray-Casting-Based Algorithm for Triangulating Surfaces at the Nanoscale. PLoS One 2013, 8 (4), e59744 10.1371/journal.pone.0059744. 23577073 PMC3618509

[ref52] Atkins P.; de Paula J. Atkin’s Physical Chemistry, 8th ed.; W. H. Freeman and Co.: New York, 2006.

[ref53] Dill K. A.; Bromberg S.; Stigter D. Molecular Driving Forces; 2nd ed.; Garland Science: 2010. 10.4324/9780203809075.

[ref54] Hill T. L. An Introduction to Statistical Thermodynamics; Addison Wesley Publishing Co.: 1960. Reprinted by Dover Publications, 1986.

[ref55] Winkler J. R.; Gray H. B. Electron Flow through Metalloproteins. Chem. Rev. 2014, 114 (7), 3369–3380. 10.1021/cr4004715. 24279515 PMC3981952

[ref56] Petrenko A.; Stein M. Rates and Routes of Electron Transfer of [NiFe]-Hydrogenase in an Enzymatic Fuel Cell. J. Phys. Chem. B 2015, 119 (43), 13870–13882. 10.1021/acs.jpcb.5b04208. 26218232

[ref57] Yan X.; Ma S.; Tang J.; Tanner D.; Ulstrup J.; Xiao X.; Zhang J. Direct Electron Transfer of Fructose Dehydrogenase Immobilized on Thiol-Gold Electrodes. Electrochim. Acta 2021, 392, 138946 10.1016/j.electacta.2021.138946.

[ref58] Ahrens J.; Geveci B.; Law C. ParaView: An End-User Tool for Large-Data Visualization. In Visualization Handbook; Elsevier: 2005; pp 717–731. 10.1016/B978-012387582-2/50038-1.

[ref59] Wittemann A.; Ballauff M. Interaction of Proteins with Linear Polyelectrolytes and Spherical Polyelectrolyte Brushes in Aqueous Solution. Phys. Chem. Chem. Phys. 2006, 8 (45), 5269 10.1039/b609879g. 19810405

[ref60] Bremer M. G. E. G.; Duval J.; Norde W.; Lyklema J. Electrostatic Interactions between Immunoglobulin (IgG) Molecules and a Charged Sorbent. Colloids Surf., A 2004, 250 (1–3), 29–42. 10.1016/j.colsurfa.2004.05.026.

[ref61] Chen K.; Xu Y.; Rana S.; Miranda O. R.; Dubin P. L.; Rotello V. M.; Sun L.; Guo X. Electrostatic Selectivity in Protein–Nanoparticle Interactions. Biomacromolecules 2011, 12 (7), 2552–2561. 10.1021/bm200374e. 21574652 PMC3134168

[ref62] Henzler K.; Haupt B.; Lauterbach K.; Wittemann A.; Borisov O.; Ballauff M. Adsorption of β-Lactoglobulin on Spherical Polyelectrolyte Brushes: Direct Proof of Counterion Release by Isothermal Titration Calorimetry. J. Am. Chem. Soc. 2010, 132 (9), 3159–3163. 10.1021/ja909938c. 20143809

[ref63] Galisteo F.; Norde W. Adsorption of Lysozyme and α-Lactalbumin on Poly(Styrenesulphonate) Latices 1. Adsorption and Desorption Behaviour. Colloids Surf., B 1995, 4 (6), 375–387. 10.1016/0927-7765(95)01181-H.

[ref64] Ninham B. W.; Parsegian V. A. Electrostatic Potential between Surfaces Bearing Ionizable Groups in Ionic Equilibrium with Physiologic Saline Solution. J. Theor. Biol. 1971, 31 (3), 405–428. 10.1016/0022-5193(71)90019-1. 5556141

[ref65] Tagliazucchi M.; Szleifer I. Stimuli-Responsive Polymers Grafted to Nanopores and Other Nano-Curved Surfaces: Structure, Chemical Equilibrium and Transport. Soft Matter 2012, 8 (28), 7292 10.1039/c2sm25777g.

[ref66] Biesheuvel P. M.; van der Veen M.; Norde W. A Modified Poisson–Boltzmann Model Including Charge Regulation for the Adsorption of Ionizable Polyelectrolytes to Charged Interfaces, Applied to Lysozyme Adsorption on Silica. J. Phys. Chem. B 2005, 109 (9), 4172–4180. 10.1021/jp0463823. 16851479

[ref67] Longo G. S.; Szleifer I. Adsorption and Protonation of Peptides and Proteins in pH Responsive Gels. J. Phys. D: Appl. Phys. 2016, 49 (32), 323001 10.1088/0022-3727/49/32/323001.

[ref68] Levin A.; Czeslik C. Interaction of Calmodulin with Poly(Acrylic Acid) Brushes: Effects of High Pressure, pH-Value and Ligand Binding. Colloids Surf., B 2018, 171, 478–484. 10.1016/j.colsurfb.2018.07.073. 30077905

[ref69] Meissner J.; Prause A.; Bharti B.; Findenegg G. H. Characterization of Protein Adsorption onto Silica Nanoparticles: Influence of pH and Ionic Strength. Colloid Polym. Sci. 2015, 293 (11), 3381–3391. 10.1007/s00396-015-3754-x. 26617429 PMC4654746

[ref70] Demanèche S.; Chapel J.-P.; Monrozier L. J.; Quiquampoix H. Dissimilar PH-Dependent Adsorption Features of Bovine Serum Albumin and α-Chymotrypsin on Mica Probed by AFM. Colloids Surf., B 2009, 70 (2), 226–231. 10.1016/j.colsurfb.2008.12.036. 19186036

[ref71] Höök F.; Rodahl M.; Kasemo B.; Brzezinski P. Structural Changes in Hemoglobin during Adsorption to Solid Surfaces: Effects of PH, Ionic Strength, and Ligand Binding. Proc. Natl. Acad. Sci. U. S. A. 1998, 95 (21), 12271–12276. 10.1073/pnas.95.21.12271. 9770476 PMC22821

[ref72] Rabe M.; Verdes D.; Seeger S. Understanding Protein Adsorption Phenomena at Solid Surfaces. Adv. Colloid Interface Sci. 2011, 162 (1–2), 87–106. 10.1016/j.cis.2010.12.007. 21295764

[ref73] Wang C.; Greene D.; Xiao L.; Qi R.; Luo R. Recent Developments and Applications of the MMPBSA Method. Frontiers in Molecular Biosciences 2018, 4, 87 10.3389/fmolb.2017.00087. 29367919 PMC5768160

[ref74] Sugimoto Y.; Kitazumi Y.; Tsujimura S.; Shirai O.; Yamamoto M.; Kano K. Electrostatic Interaction between an Enzyme and Electrodes in the Electric Double Layer Examined in a View of Direct Electron Transfer-Type Bioelectrocatalysis. Biosens. Bioelectron. 2015, 63, 138–144. 10.1016/j.bios.2014.07.025. 25078712

[ref75] Sugimoto Y.; Kitazumi Y.; Shirai O.; Yamamoto M.; Kano K. Role of 2-Mercaptoethanol in Direct Electron Transfer-Type Bioelectrocatalysis of Fructose Dehydrogenase at Au Electrodes. Electrochim. Acta 2015, 170, 242–247. 10.1016/j.electacta.2015.04.164.

[ref76] Utesch T.; Millo D.; Castro M. A.; Hildebrandt P.; Zebger I.; Mroginski M. A. Effect of the Protonation Degree of a Self-Assembled Monolayer on the Immobilization Dynamics of a [NiFe] Hydrogenase. Langmuir 2013, 29 (2), 673–682. 10.1021/la303635q. 23215250

[ref77] Singh K.; McArdle T.; Sullivan P. R.; Blanford C. F. Sources of Activity Loss in the Fuel Cell Enzyme Bilirubin Oxidase. Energy Environ. Sci. 2013, 6 (8), 2460 10.1039/c3ee00043e.

[ref78] Benavidez T. E.; Torrente D.; Marucho M.; Garcia C. D. Adsorption of Soft and Hard Proteins onto OTCEs under the Influence of an External Electric Field. Langmuir 2015, 31 (8), 2455–2462. 10.1021/la504890v. 25658387 PMC4433030

[ref79] Baggioli A.; Casalegno M.; Raos G.; Muccioli L.; Orlandi S.; Zannoni C. Atomistic Simulation of Phase Transitions and Charge Mobility for the Organic Semiconductor Ph-BTBT-C10. Chem. Mater. 2019, 31 (17), 7092–7103. 10.1021/acs.chemmater.9b02882.

[ref80] Heidary N.; Utesch T.; Zerball M.; Horch M.; Millo D.; Fritsch J.; Lenz O.; Von Klitzing R.; Hildebrandt P.; Fischer A.; Mroginski M. A.; Zebger I. Orientation-Controlled Electrocatalytic Efficiency of an Adsorbed Oxygen-Tolerant Hydrogenase. PLoS One 2015, 10 (11), e0143101 10.1371/journal.pone.0143101. 26580976 PMC4651547

[ref81] Hexter S. V.; Esterle T. F.; Armstrong F. A. A Unified Model for Surface Electrocatalysis Based on Observations with Enzymes. Phys. Chem. Chem. Phys. 2014, 16 (24), 11822–11833. 10.1039/c3cp55230f. 24556983

[ref82] Aragon S. R. Recent Advances in Macromolecular Hydrodynamic Modeling. Methods 2011, 54 (1), 101–114. 10.1016/j.ymeth.2010.10.005. 21073955 PMC3085554

[ref83] Ciaccafava A.; Infossi P.; Ilbert M.; Guiral M.; Lecomte S.; Giudici-Orticoni M. T.; Lojou E. Electrochemistry, AFM, and PM-IRRA Spectroscopy of Immobilized Hydrogenase: Role of a Hydrophobic Helix in Enzyme Orientation for Efficient H2 Oxidation. Angew. Chem., Int. Ed. 2012, 51 (4), 953–956. 10.1002/anie.201107053. 22173906

[ref84] Sedenho G. C.; Hassan A.; de Souza J. C. P.; Crespilho F. N. In Situ and Operando Electrochemistry of Redox Enzymes. Current Opinion in Electrochemistry 2022, 34, 101015 10.1016/j.coelec.2022.101015.

