# Supplementary material for: Modeling of the Electrostatic Interaction and Catalytic Activity of [NiFe] Hydrogenases on a Planar Electrode
Source: J Phys Chem B. 2022 Oct 21;126(43):8777–90. doi: 10.1021/acs.jpcb.2c05371 (PMC9639099; doi:10.1021/acs.jpcb.2c05371)
Supplement: Supplementary file 1 — jp2c05371_si_001.zip [file jp2c05371_si_001.zip › Supplementary data/Hydrogenase Adsorption - Support Information.pdf]

Supplementary Information for the J. Phys. Chem. B. manuscript

Modeling of the electrostatic interaction and catalytic activity of [NiFe] hydrogenases on a planar electrode

by Manuel Antonio Ruiz-Rodríguez, Christopher D. Cooper, Walter Rocchia, Mosè Casalegno, Yossef López - de los Santos and Guido Raos.

Electron transfer rates

Table SI1 collects the electron transfer rates calculated by Petrenko and Stein ["Rates and Routes of Electron Transfer of [NiFe]-Hydrogenase in an Enzymatic Fuel Cell". *The Journal of Physical Chemistry B* **2015**, *119* (43), 13870–13882. https://doi.org/10.1021/acs.jpcb.5b04208]. Figure SI1 shows a fit of the data according to the exponential decay of Eq. (19). The fit yields the value β=0.45 Å^{-1} used in our calculations.

Table S1. Rates of electron transfer (*k*) as a function of the distance between the external iron-sulfur cluster and a graphite electrode (*Z*), as calculated by Petrenko and Stein.

Z (Å)	k (s^{-1})	ln(k)
12.3	5100	8.54
28.3	4.5	1.50
24.9	22	3.09
15.3	2200	7.70

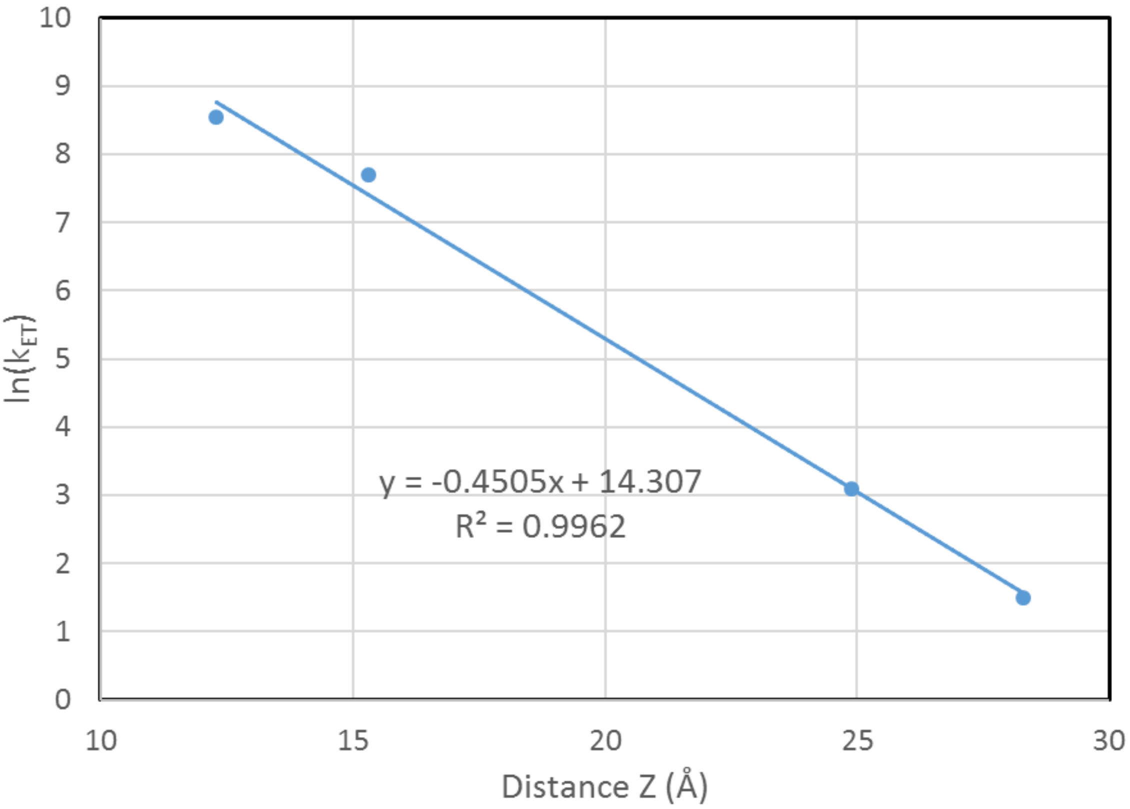

Figure S1. Fit of the data in Table SI1.

Reference current densities

Table SI2 and Figure SI2 compare the reference current densities J_0, calculated according Eq. (21) with two different β values. The two sets of data have been rescaled independently, so that the maximum value of J_0 is equal to 1.00 in each case. Notice that there is good correlation between the data for low current densities, but the two models lead to different predictions for the system with the largest current density.

Table SI2. Reference current densities, for different β values.

	pH	J_0 (a.u.), β=0.45 Å^{-1}	J_0 (a.u.), β=1.40 Å^{-1}
I=0.15 M, [illegible]$_e$=-0.05 V	5	0.350	0.232
	6	0.507	0.645
	7	0.482	1.000
	8	0.420	0.549
	9	0.368	0.341
I=0.15 M, [illegible]$_e$=0.0 V	5	0.481	0.454
	6	0.421	0.341
	7	0.385	0.491
	8	0.403	0.477
	9	0.433	0.600
I=0.15 M, [illegible]$_e$=+0.05 V	5	0.612	0.522
	6	0.517	0.578
	7	0.477	0.752
	8	0.427	0.536
	9	0.498	0.914
I=0.00 M, [illegible]$_e$=0.0 V	5	1.000	0.581
	6	0.656	0.399
	7	0.324	0.615
	8	0.153	0.299
	9	0.082	0.113

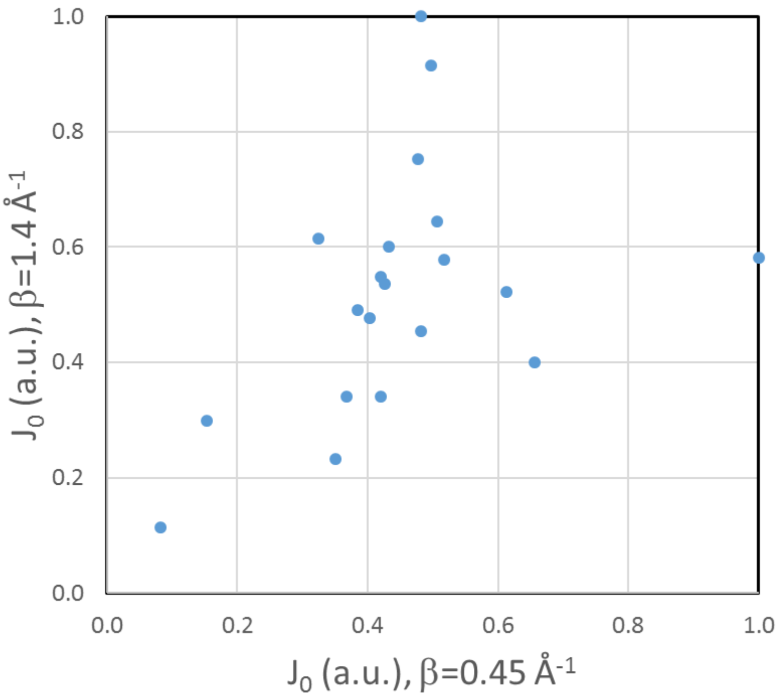

Figure SI2. Scatter plot of the data in Table SI2.
