# Supplementary material for: Modeling of the Electrostatic Interaction and Catalytic Activity of [NiFe] Hydrogenases on a Planar Electrode
Source: J Phys Chem B. 2022 Oct 21;126(43):8777–90. doi: 10.1021/acs.jpcb.2c05371 (PMC9639099; doi:10.1021/acs.jpcb.2c05371)
Supplement: Supplementary file 1 — jp2c05371_si_001.zip [file jp2c05371_si_001.zip › Supplementary data/README.docx]

README

The supplementary data contains:

1. Two examples ready to be run in PyGBe: the orientations 1e3d_5_theta_40_phi_98 and 1e3d_5_theta_120_phi_248 with their respective inputs and outputs, inside of the folders there are the “.pqr” files, the meshes, the “.config” and “.param” files that have the instruction for the calculations in PyGBe.

To execute a run in PyGBe type in the terminal PyGBe + folder name, example: pygbe 1e3d_5_theta_40_phi_98.

2. A folder named “Metallic clusters orca” with the inputs and outputs for the calculations of the atomic charges used for “pdb2pqr” ready to be used for “ORCA”.

3. The AMBER modified file created from the results of the atomic charges calculated with “ORCA”.

4. A script of python named “vtk_generator_stern_gr.py” used to generate the “.vtk files” used for the visualization of the results of PyGBe. These files can be opened with “Paraview”.

5. A pdf file named “Hydrogenase Adsorption - Support Information.pdf” containing data and plots related to the electron transfer between protein and electrode.
