# Supplementary material for: Modeling of the Electrostatic Interaction and Catalytic Activity of [NiFe] Hydrogenases on a Planar Electrode
Source: J Phys Chem B. 2022 Oct 21;126(43):8777–90. doi: 10.1021/acs.jpcb.2c05371 (PMC9639099; doi:10.1021/acs.jpcb.2c05371)
Supplement: Supplementary file 1 — jp2c05371_si_001.zip [file jp2c05371_si_001.zip › Supplementary data/~WRL1100.tmp]

README

The supplementary data contains:

1. Two examples ready to be run in pygbe: the orientations 1e3d_5_theta_40_phi_98 and 1e3d_5_theta_120_phi_248 with their respective inputs and outputs, inside of the folders there are the “.pqr” files, the meshes, the “.config” and “.param” files that have the instruction for the calculations in pygbe.

To execute a run in pybe type in the terminal pygbe + folder’name, example: pygbe 1e3d_5_theta_40_phi_98

2. A folder named “Metallic clusters orca” with the inputs and outputs for the calculations of the charges ready to be used for “ORCA”

3. The AMBER modified files created from the results of the atomic charges obtained with “ORCA”

4. An script of python named “vtk_generator_stern_gr.py” to generate the “.vtk files” used for the visualization of the results of pygbe. These files need to be open with “Paraview”
