# Supplementary figures and images for: Modeling of the Electrostatic Interaction and Catalytic Activity of [NiFe] Hydrogenases on a Planar Electrode
Source: J Phys Chem B. 2022 Oct 21;126(43):8777–90. doi: 10.1021/acs.jpcb.2c05371 (PMC9639099; doi:10.1021/acs.jpcb.2c05371)

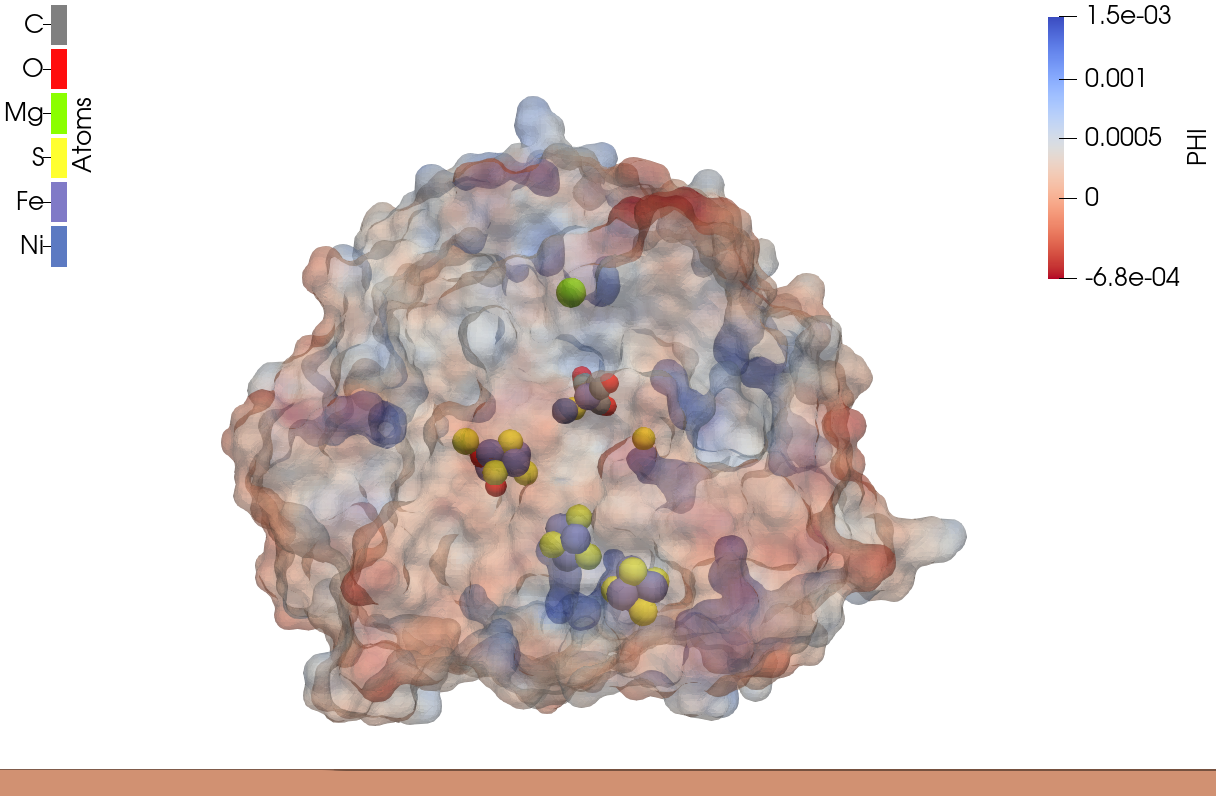

Supplement: Supplementary file 1 — jp2c05371_si_001.zip [file jp2c05371_si_001.zip › Supplementary data/1e3d_5_theta_120_phi_248/extra information/1e3d_5_theta_120_phi_248-5.tiff]

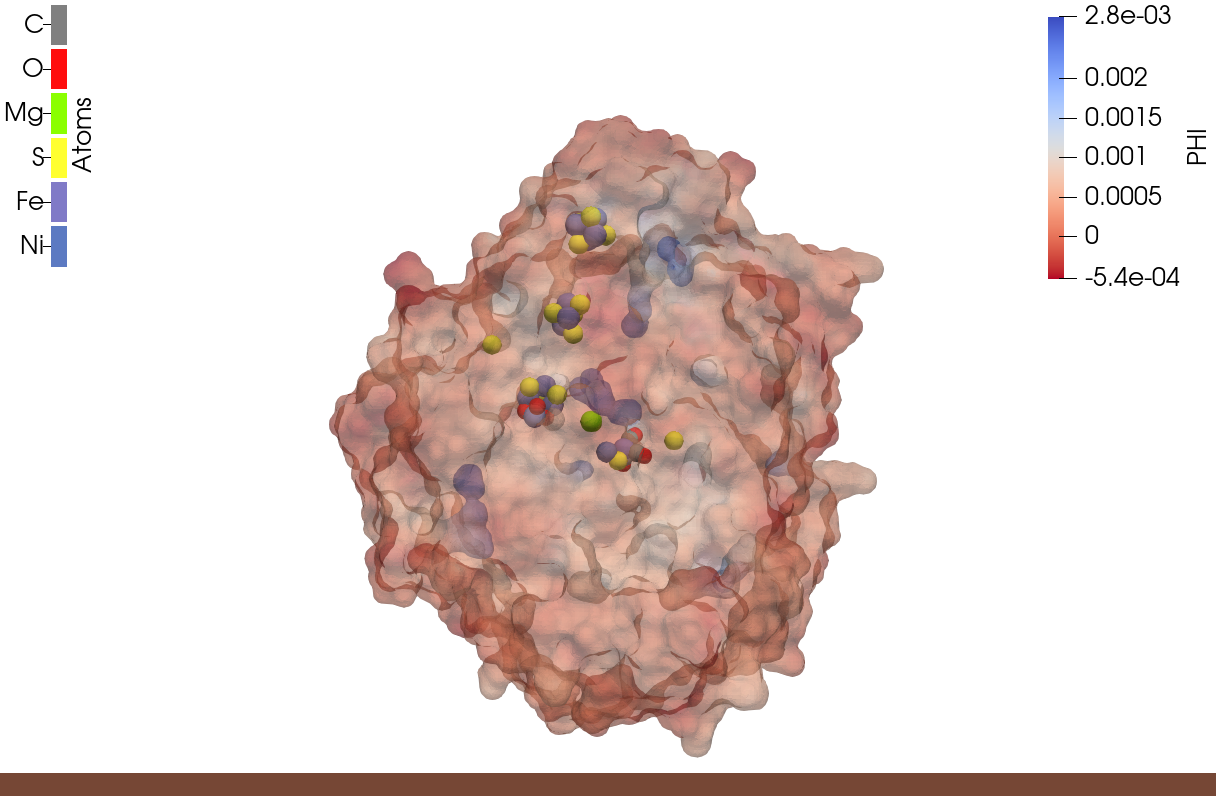

Supplement: Supplementary file 1 — jp2c05371_si_001.zip [file jp2c05371_si_001.zip › Supplementary data/1e3d_5_theta_40_phi_98/extra information/1e3d_5_theta_40_phi_98-5.tiff]
